# Integrating multi-modal biological knowledge via contrastive dual-view graph learning for phosphorylation site-disease association prediction

**DOI:** 10.1093/bioinformatics/btag690

**Published:** 2026-09-18

**Authors:** Xiangyu Chen, Lijun Quan, Yexuan Mao, Siyuan Wang, Guozheng Zhang, Siqi Li, Yelu Jiang, Liangpeng Nie, Tingfang Wu, Qiang Lyu

**Affiliations:** School of Computer Science and Technology, Soochow University, Suzhou 215006, China; School of Computer Science and Technology, Soochow University, Suzhou 215006, China; Collaborative Innovation Center of Novel Software Technology and Industrialization, Nanjing 210023, China; Jiangsu Key Laboratory of Drug Discovery and Translational Research for Brain Diseases, Soochow University, Suzhou 215123, China; School of Computer Science and Technology, Soochow University, Suzhou 215006, China; School of Computer Science and Technology, Soochow University, Suzhou 215006, China; School of Computer Science and Technology, Soochow University, Suzhou 215006, China; School of Computer Science and Technology, Soochow University, Suzhou 215006, China; School of Computer Science and Technology, Soochow University, Suzhou 215006, China; School of Computer Science and Technology, Soochow University, Suzhou 215006, China; School of Computer Science and Technology, Soochow University, Suzhou 215006, China; Collaborative Innovation Center of Novel Software Technology and Industrialization, Nanjing 210023, China; Jiangsu Key Laboratory of Drug Discovery and Translational Research for Brain Diseases, Soochow University, Suzhou 215123, China; School of Computer Science and Technology, Soochow University, Suzhou 215006, China; Collaborative Innovation Center of Novel Software Technology and Industrialization, Nanjing 210023, China; Jiangsu Key Laboratory of Drug Discovery and Translational Research for Brain Diseases, Soochow University, Suzhou 215123, China

## Abstract

**Motivation:**

Accurately characterizing the associations between phosphorylation sites (psites) and diseases is essential for elucidating pathogenic mechanisms and guiding therapeutic discovery. However, existing computational approaches for phosphorylation site–disease association (PDA) prediction remain limited, as they often overlook the integration of biological knowledge across multiple modalities.

**Results:**

We propose CDVGL-PDA, a contrastive dual-view graph learning framework for PDA prediction. Specifically, we construct a multi-modal heterogeneous graph encompassing nine node types and ten edge types, enabling comprehensive representation of phosphorylation-centric biological networks. For phosphorylation site nodes, CDVGL-PDA incorporates sequence-derived embeddings, while disease nodes are initialized with semantic features from BioBERT. The framework then performs dual-view heterogeneous graph encoding, aligns representations through contrastive learning, and adaptively integrates them via attention-based fusion to capture informative embeddings. Extensive evaluations demonstrate CDVGL-PDA’s strong predictive performance across balanced, imbalanced, and low-similarity datasets. Analysis of embeddings reveals that the model effectively captures latent biological relationships. Ablation and visualization studies validate the contributions of each module, while case studies highlight its ability to uncover potential PDAs, illustrating its promise for advancing disease mechanism research and therapeutic target discovery.

**Availability and Implementation:**

The source code and datasets for CDVGL-PDA are publicly available in the GitHub repository at https://github.com/ljquanlab/CDVGL-PDA/.

## 1 Introduction

Post-translational modifications (PTMs) are chemical modifications occurring after protein translation, including phosphorylation, acetylation, and ubiquitination (Ye *et al.* 2022). They play essential roles in regulating protein function, stability, localization, and interactions, and are central to key biological processes such as signal transduction, metabolism, and gene expression (Leutert *et al.* 2021). Among them, protein phosphorylation is one of the most extensively studied PTMs, broadly involved in cellular signaling and physiological regulation (Mann *et al.* 2002). It mainly occurs on serine, threonine, and tyrosine residues, catalyzed by kinases that add phosphate groups to amino acid side chains, thereby modulating protein conformation, activity, and interactions (Olsen *et al.* 2006).

Aberrant phosphorylation has been widely implicated in diverse diseases. For instance, Tau hyperphosphorylation promotes neurofibrillary tangle formation in Alzheimer disease (Chakraborty *et al.* 2024), while dysregulated phosphorylation and O-GlcNAcylation of troponin T are associated with ischaemic heart failure (Dubois-Deruy *et al.* 2015). In addition, elevated phosphorylation of Hsp27 at Ser78 is linked to HER-2/neu-positive breast cancer progression and metastasis (Zhang *et al.* 2007). These findings suggest that phosphorylation sites (psites) are not only potential biomarkers but also functional contributors to disease pathogenesis. Therefore, systematic identification of phosphorylation site–disease associations (PDAs) is critical for understanding molecular mechanisms and facilitating biomarker discovery and targeted therapy development.

To date, computational studies have mainly focused on identifying protein psites (Pham *et al.* 2024, Sabir *et al.* 2024) and predicting functional psites (Zhang *et al.* 2024, Xie *et al.* 2025), whereas computational studies on PDAs remain limited. Early efforts employed traditional machine learning and network-based methods. For example, Xu *et al.* (2015) proposed an SVM-based feature selection framework to identify disease-specific psites, and later developed a multilayer heterogeneous network with random walk for PDA prediction (Xu and Wang 2015). He *et al.* (2022) applied deep convolutional neural networks with transfer learning to predict leukemia-related psites. More recently, Deng *et al.* (2024) integrated multiple similarity networks and random walk with restart, followed by a convolutional neural network, to identify potential PDAs.

Existing computational approaches primarily rely on traditional machine learning methods or random walk-based graph algorithms. However, these methods often struggle to capture the complex and heterogeneous relationships inherent in biological systems. In recent years, heterogeneous graph neural networks have been widely applied to various graph-structured tasks and have demonstrated strong performance in modeling complex biological systems, such as drug-target interaction prediction (Song *et al.* 2024, Zhang *et al.* 2025). By integrating multiple types of nodes and their rich semantic relationships, heterogeneous graphs enable more comprehensive representations of biological entities. Therefore, leveraging heterogeneous graph frameworks to integrate multi-source biological data holds great potential for improving the prediction of PDAs.

In this study, we propose a contrastive dual-view graph learning framework, CDVGL-PDA, for predicting PDAs. We first construct a heterogeneous graph integrating multiple biological modalities, encompassing phosphorylation site (psite) sequence information, protein sequence information, biomedical text semantics, and graph-based biological knowledge, resulting in a heterogeneous network with nine node types and ten edge types. Node features are initialized using modality-specific encoders. The graph is then processed by two heterogeneous graph encoders, RGAT and HGT, to learn complementary node representations by jointly modeling attributes and topology. A contrastive learning strategy is employed to align representations of the same nodes across views, enhancing discriminative power, followed by an attention-based fusion mechanism to integrate multi-view features. Finally, association scores between psites and diseases are computed using the ComplEx scoring function.

Experimental results show that CDVGL-PDA consistently outperforms baseline methods across benchmarks with varying class imbalance ratios and remains robust under low-similarity settings. Embedding analyses further demonstrate its ability to capture disease relationships, while case studies on Alzheimer disease and colorectal neoplasms validate its practical applicability. Ablation and interpretability analyses highlight the contributions of key modules and provide insights into model behavior.

## 2 Results

### 2.1 Overview of CDVGL-PDA framework

The overall framework of CDVGL-PDA is shown in Fig. 1a. We first construct a heterogeneous biological graph consisting of nine node types and ten edge types (Fig. 1b and Table 1). PDAs are not included as edges in the graph topology, and the graph encodes auxiliary biological associations and serves as a contextual knowledge network for representation learning. For node feature initialization, psite features are extracted from primary amino acid sequences using a dedicated sequence encoder (Fig. 1c), while disease features are obtained by encoding disease names using BioBERT. Other node types are initialized using their corresponding biological attributes. Then, the graph is processed by two heterogeneous graph encoders, RGAT and HGT, to learn complementary node representations by jointly modeling node features and graph topology. A contrastive learning objective is applied during training to align node representations across the two views. The resulting representations are further integrated using an attention-based fusion mechanism to obtain unified node embeddings. Finally, a scoring function based on ComplEx is employed to estimate the association probability between psite-disease pairs. For each candidate pair, the corresponding node embeddings are retrieved and fed into the scoring function, producing a matching score, i.e. used for binary classification of PDA associations. Model hyperparameters were selected through systematic search and validation. Detailed settings are provided in Supplementary Information (SI) Text S1.

**Figure 1:**
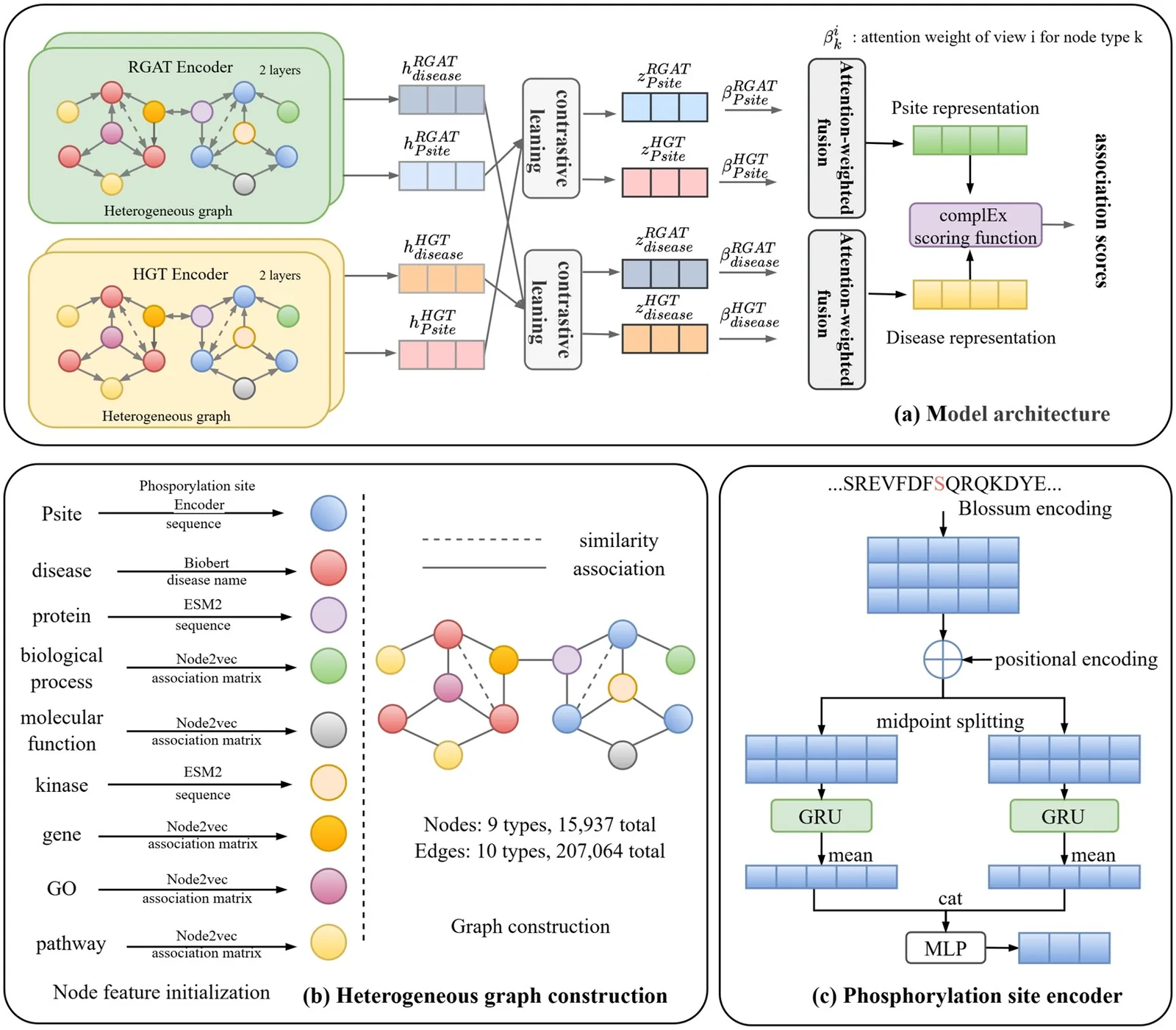
The overall architecture of CDVGL-PDA. (a) The model architecture of CDVGL-PDA. The heterogeneous graph is processed in parallel by two graph encoders (HGT and RGAT). Contrastive learning maximizes agreement between the two views of each node, and an attention-weighted fusion mechanism integrates the dual-view representations into unified psite and disease node representations. ComplEx is then used to compute association scores between psites and diseases. (b) Heterogeneous graph construction. The heterogeneous graph consists of nine types of nodes and ten types of edges. (c) Phosphorylation site encoder. Two bidirectional GRUs are used to capture the contextual information of psite, and the updated psite node embedding is obtained through averaging and concatenation operations.

**Table 1:** Statistics of node and edge types in the heterogeneous graph.

Node		Edge	
Type	Number	Type	Number
GO	3657	disease–disease	1550
disease	293	gene–disease	12 979
molecular function	28	GO–disease	77 524
gene	5579	pathway–disease	89 173
kinase	517	gene–protein	593
pathway	2261	molecular function–psite	4574
psite	2322	kinase–psite	4130
biological process	47	biological process–psite	6011
protein	1233	protein–psite	2322
		psite–psite	8208
Total	15 937	Total	207 064

### 2.2 Predictive performance under different class distributions

To evaluate model performance under different class distributions, we tested CDVGL-PDA on benchmark datasets with varying positive-to-negative ratios, namely Balance-1to1, Imbalance-1to5, and Imbalance-1toall, as described in Section 3.2. On the Balance-1to1 benchmark using 10-fold cross-validation, CDVGL-PDA achieved an average AUC of 0.9515, an average AUPR of 0.9562, and an F1 score of 0.8892. Compared with the second-best method, CDVGL-PDA yields relative improvements of 1.29% in AUC, 1.0% in AUPR, and 2.6% in F1 score, as detailed in SI Table S1. CDVGL-PDA achieves competitive performance across these metrics, indicating generally good discrimination.

Ten-fold cross-validation was conducted on the Imbalance-1to5 and Imbalance-1toall benchmarks, showing that CDVGL-PDA consistently outperforms all baseline methods (Fig. 2a). Specifically, on the Imbalance-1to5 benchmark, CDVGL-PDA achieves average AUC, AUPR, and F1 scores of 0.9695, 0.9320, and 0.8732, respectively, corresponding to relative improvements of approximately 1.96%, 5.25%, and 8.17% (SI Table S2). On the more challenging Imbalance-1toall benchmark, CDVGL-PDA achieves an average AUC of 0.9718, AUPR of 0.8593, and F1 score of 0.8505, with relative improvements of about 0.9%, 8.63%, and 24.94% (SI Table S3). These results indicate that CDVGL-PDA not only demonstrates strong discriminative ability but also maintains stable and even more pronounced performance advantages as class imbalance becomes more severe.

**Figure 2:**
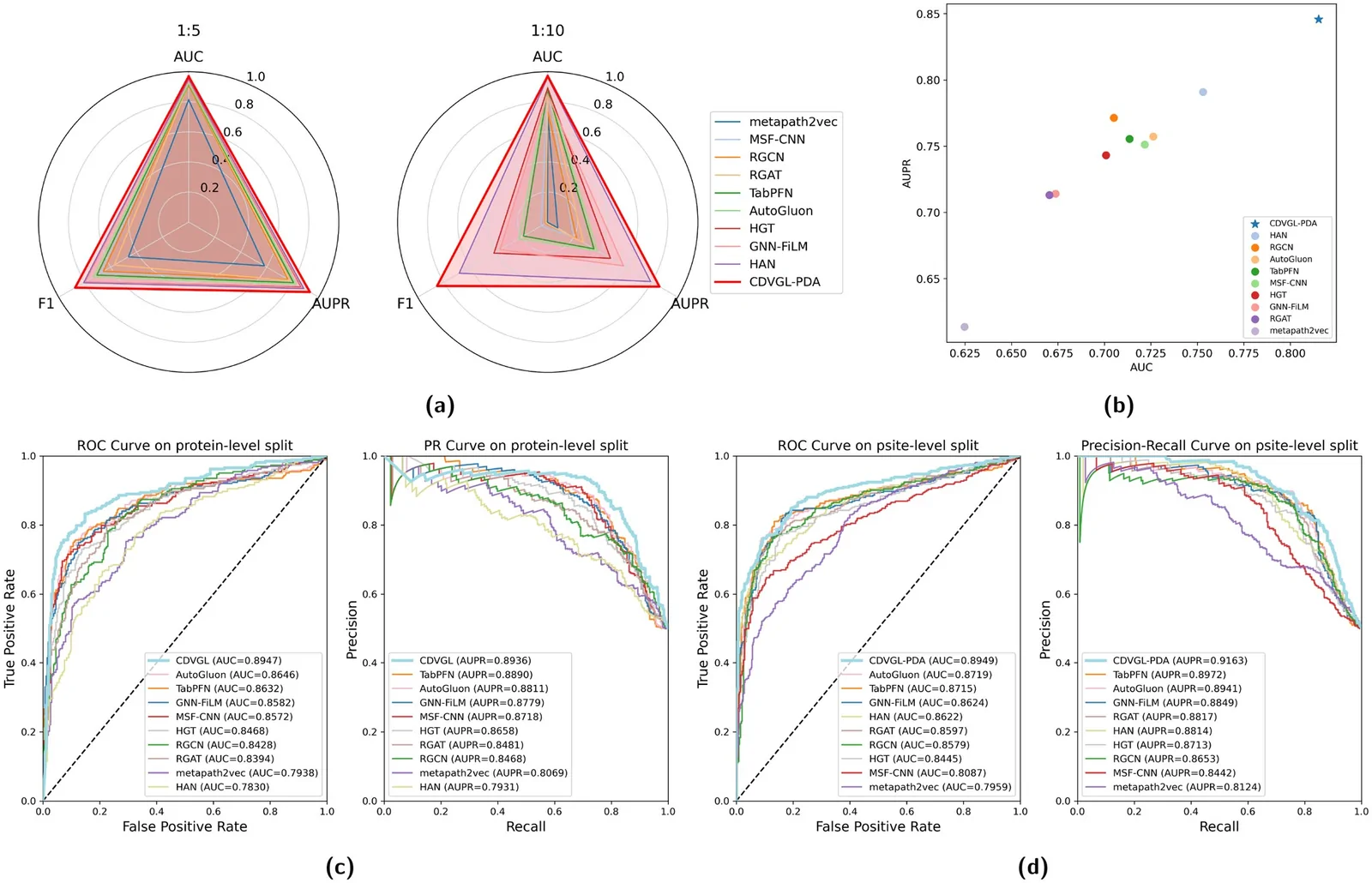
Performance comparison between CDVGL-PDA and baseline methods under class-imbalanced and low-similarity settings. (a) Results on imbalanced benchmarks with ratios of 1:5 and 1: all, evaluated by AUC, AUPR, and F1-score. (b) Performance comparison on the pair-level split benchmark. (c) Performance comparison on the protein-level split benchmark. (d) Performance comparison on the psite-level split benchmark.

To further examine whether the observed performance gains are statistically reliable, we conducted a comparison between CDVGL-PDA and the second-best method using the 10-fold cross-validation results from all three benchmarks. Specifically, we applied a correlated Bayesian paired t-test, a hierarchical statistical modeling approach, i.e. well-suited for cross-validation-based evaluation, to estimate the posterior probability that CDVGL-PDA outperforms the second-best method, and we also computed the corresponding *P* values using the conventional paired *t*-test. Across all settings, CDVGL-PDA exhibits posterior probabilities exceeding 95% that it outperforms the second-best method in both AUPR and AUC, with paired *t*-test *P* values below .01 (SI Table S4). These results provide statistical support for the consistent improvements observed across all class imbalance scenarios.

### 2.3 Predictive performance on low-similarity benchmarks

To assess generalization to low-similarity space, we evaluated CDVGL-PDA on three low-similarity benchmarks, namely protein-level split, psite-level split, and pair-level split, as described in Section 3.2. On the pair-level split, where both psite and disease similarities between training and test pairs are explicitly minimized, CDVGL-PDA achieved an AUC of 0.8151 and an AUPR of 0.8458, corresponding to relative improvements of approximately 8.24% and 6.94% over the second-best method (see Fig. 2b, SI Table S5). On the protein-level split, CDVGL-PDA achieved an AUC of 0.8947 and an AUPR of 0.8936, corresponding to relative improvements of approximately 3.48% and 0.51% over the respective second-best methods (see Fig. 2c, SI Table S5). On the psite-level split, CDVGL-PDA obtained an AUC of 0.8949 and an AUPR of 0.9163, yielding relative gains of about 2.63% and 2.12% over the respective second-best methods (see Fig. 2d, SI Table S5).

Although the variation in performance across the low-similarity splits is limited, CDVGL-PDA consistently achieves meaningful predictive results. These low-similarity evaluations reduce potential overlap between training and test data, thereby providing a more reliable estimate of model performance. The results show that CDVGL-PDA effectively captures both network topology and local sequence context, producing transferable embeddings that generalize to distinct psite contexts. In addition, further analyses of prediction performance stratified by node degree suggest that CDVGL-PDA maintains reasonable performance across nodes with different connectivity levels, including low-degree cases (details in SI Text S2 and SI Table S6).

### 2.4 Capturing relationships among diseases by CDVGL-PDA

We computed cosine similarities between learned disease embeddings and compared them with MeSHDAG-based semantic similarities (details in SI Text S3). The MeSHDAG-based matrix is highly sparse (average similarity 0.02), reflecting limited shared hierarchy, whereas the embedding-based matrix shows a continuous distribution (average 0.61), indicating broader cross-category similarity (SI Fig. S2). Correlation analysis (Fig. 3a) reveals a weak but positive association (Pearson’s r=0.343, Spearman’s ρ=0.549), suggesting that embeddings partially capture MeSH hierarchy while not being solely determined by it.

**Figure 3:**
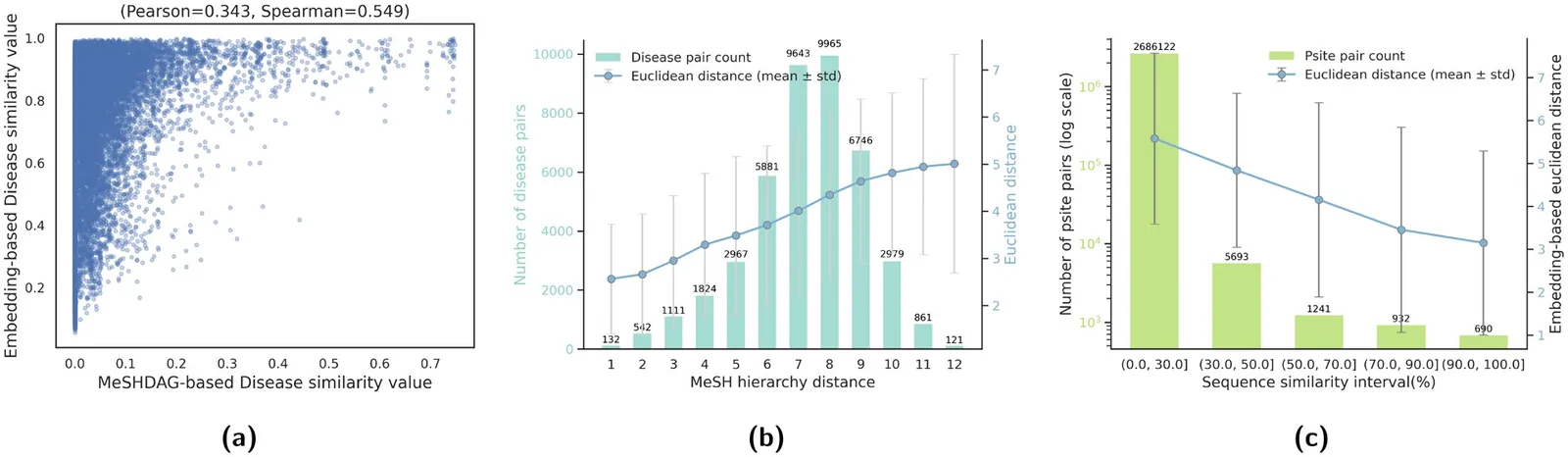
Analysis of biological consistency in learned embeddings. (a) Positive correlation between embedding-based disease similarity and MeSHDAG-based disease similarity. (b) Disease pairs show a positive correlation between MeSH hierarchical distance and embedding distance, where more distant nodes in the MeSH hierarchy correspond to larger embedding distances. (c) Psite pairs exhibit a correlation between sequence similarity and embedding distance, where more similar sequences correspond to smaller embedding distances.

To further assess this, we examined disease pairs with low MeSH similarity but high embedding similarity. Over 85% of such pairs share common neighbors in the heterogeneous network, with the proportion increasing as embedding similarity rises (SI Table S7). This indicates that diseases distant in the MeSH hierarchy can still be functionally related through shared molecular context. For example, Hypertension-Inflammation shows zero MeSH similarity but high embedding similarity (0.89), sharing numerous neighbors (636 GO terms, 567 pathways, and 41 genes), consistent with evidence linking inflammation to hypertension pathogenesis (Xiao and Harrison 2020), where clinical studies have shown that hypertension is often accompanied by chronic low-grade inflammation, characterized by elevated plasma C-reactive protein and multiple inflammatory factors, with immune and inflammatory pathways playing key roles in its pathogenesis.

Further analyses show that removing disease-disease edges does not eliminate hierarchical patterns in the learned embeddings (Fig. 3b, details in SI Text S4), suggesting that such relationships can be inferred from heterogeneous context. Similar observations hold for psites, where sequence similarity is preserved after removing psite-psite edges (see Fig. 3c, details in SI Text S5). In addition, disease similarity is positively correlated with shared psites (details in SI Text S6 and SI Fig. S3), further supporting the biological relevance of the learned representations. Overall, these results demonstrate that CDVGL-PDA integrates heterogeneous information to capture biologically meaningful relationships.

### 2.5 Case study

To evaluate the practical applicability of our model, we conducted case studies on colorectal neoplasms (CRC) and Alzheimer disease (AD). For each disease, we selected the top 10 psites ranked by predicted association scores. For AD, all known AD-psite associations were first removed, and the remaining PDAs, along with an equal number of randomly sampled negatives, were used for training. AD was used as the target disease and paired with all candidate psites to obtain association scores from the trained model. The scores were ranked in descending order, and the top 10 psites were selected. The same procedure was applied to CRC. Finally, the predicted PDAs were validated using PSP and PTMD 2.0.

AD is a progressive neurodegenerative disorder characterized by memory loss and cognitive decline, with hallmark pathological features including β-amyloid plaques and tau neurofibrillary tangles (Knopman *et al.* 2021). Among the top 10 predicted AD-related psites, nine are supported by existing evidence in PSP and PTMD 2.0 (see Table 2). Notably, phosphorylation at the T217 site has been confirmed by multiple studies to be relevant to AD development, e.g. Sato *et al.* (2021) found that its phosphorylation level is closely associated with hyperphosphorylated tau in cerebrospinal fluid, suggesting its potential as an early biomarker. However, the association between AD and P02730_Y359 remains to be further validated.

**Table 2:** Top 10 predicted protein psites associated with Alzheimer disease.

Rank	Psite	Evidence
1	P10636-8_T217	PTMD2.0, PSP
2	P10636-8_T212	PTMD2.0, PSP
3	P10636-8_T181	PTMD2.0, PSP
4	P10636-8_T205	PTMD2.0, PSP
5	P10636-8_S202	PTMD2.0, PSP
6	P10636-8_S262	PTMD2.0, PSP
7	P10636-8_S214	PTMD2.0, PSP
8	P10636-8_S199	PTMD2.0, PSP
9	P02730_Y359	Unconfirmed
10	P10636-8_S422	PTMD2.0, PSP

CRC is a common malignant tumor occurring in the colon or rectum, often arising from adenomatous polyps and associated with high incidence and mortality worldwide (Hossain *et al.* 2022). All of the top 10 predicted CRC-related psites are validated by PSP and PTMD 2.0, indicating strong biological relevance (see Table 3). For instance, RIOK1 phosphorylates G3BP2(Q9UN86) at Thr226, and this modification has been reported to promote p53 ubiquitination and radioresistance in CRC, supporting the disease relevance of this psite (Chen *et al.* 2022). In addition, a complete list of high-confidence, previously unconfirmed AD- and CRC-related psite associations predicted by CDVGL-PDA is provided in SI Tables S28 and S29 to facilitate future experimental validation. Furthermore, benchmark comparisons on AD and CRC further demonstrate the effectiveness of our model (see SI Text S7 and SI Fig. S4).

**Table 3:** Top 10 predicted protein psites associated with colorectal neoplasms.

Rank	Psite	Evidence
1	Q9UN86_T226	PTMD2.0, PSP
2	O14744_S15	PTMD2.0, PSP
3	O14757_S317	PTMD2.0, PSP
4	P01100_S374	PTMD2.0, PSP
5	P10070_T1546	PTMD2.0, PSP
6	Q96AC1_S159	PTMD2.0, PSP
7	Q15910_T487	PTMD2.0, PSP
8	Q9Y6A5_S558	PTMD2.0, PSP
9	P01106_S77	PTMD2.0, PSP
10	P00558_S203	PTMD2.0, PSP

### 2.6 Visualization and interpretability of CDVGL-PDA

To better understand how CDVGL-PDA extracts informative signals from the heterogeneous biological network, we analyse model interpretability from both global and local perspectives. As shown in Fig. 4a and b, the model effectively captures meaningful semantic contributions of heterogeneous relations at a global level and adaptively assigns attention to important neighboring nodes at a local level. Detailed analyses are provided in SI Text S8.

**Figure 4:**
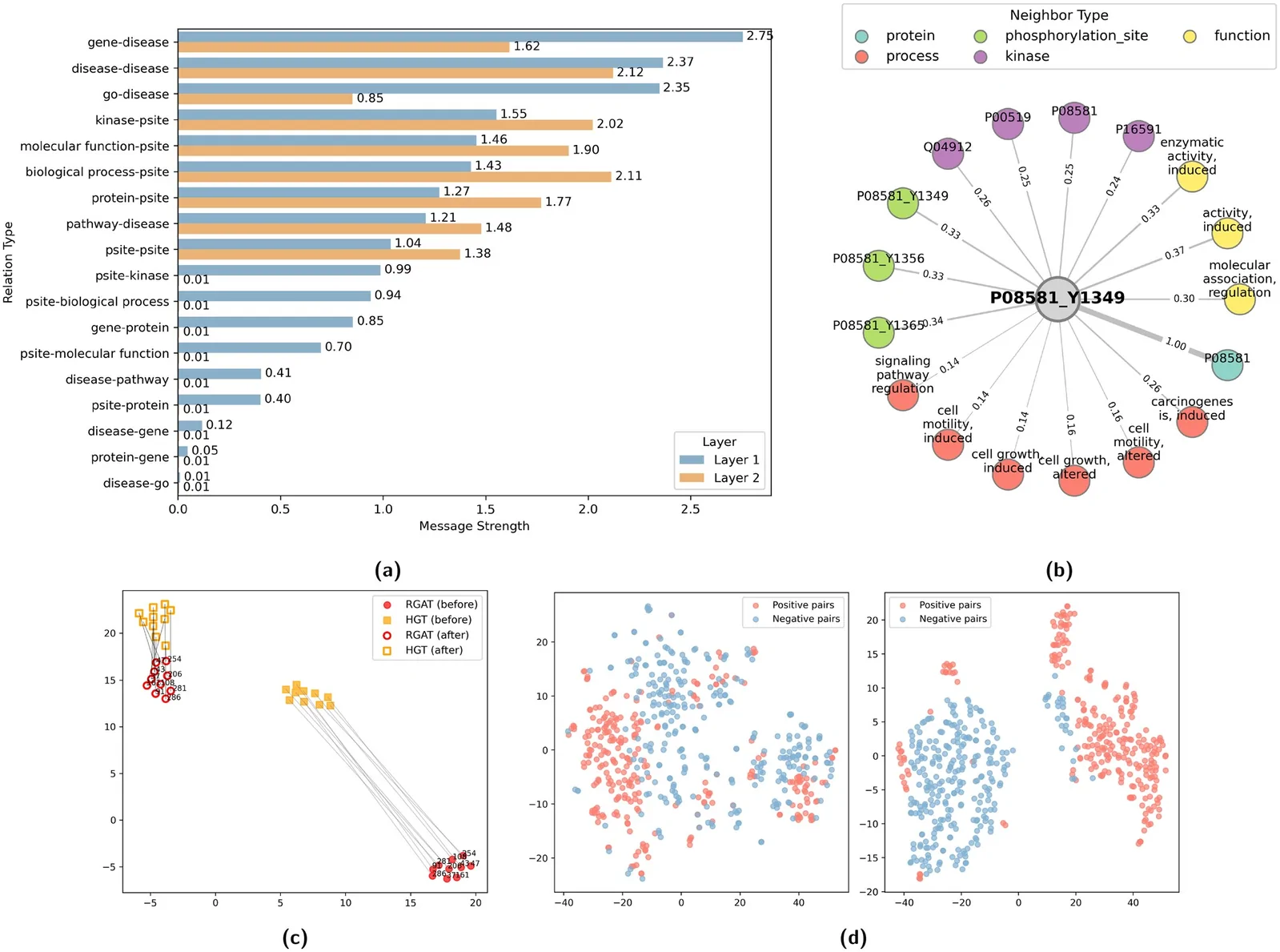
The interpretability analyses of CDVGL-PDA. (a) Global semantic importance of different relations. This figure illustrates the information propagation strength of different meta-relations across two HGT layers, reflecting the model’s overall emphasis on distinct relational channels during global semantic modeling. Relations targeting psite or disease nodes (e.g. kinase–psite and pathway–disease) are substantially strengthened in the second layer. (b) Local attention analysis of a representative psite during RGAT aggregation. Neighbor nodes are grouped by relation type, and edge widths indicate their contribution to updating the target psite. (c) Visualization of disease embeddings associated with P29317_S897 before and after contrastive learning. (d) Visualization of joint embeddings of positive and negative PDAs before and after training by CDVGL-PDA.

To evaluate the effectiveness of the contrastive learning module, we selected diseases associated with P29317_S897 from the psite-level test set and extracted their corresponding embeddings. UMAP (McInnes *et al.* 2018) was used to visualize embeddings before and after contrastive learning, where solid and hollow points denote embeddings before and after optimization, respectively, and colors indicate different views. Corresponding diseases across views are connected by lines. As shown in Fig. 4c, distances between paired embeddings from different views are markedly reduced after contrastive learning. Similar patterns are observed for psite embeddings (SI Fig. S5). Quantitatively, the average Euclidean distance between disease embeddings decreases from 1.405 to 0.607 (56.8% reduction), while that of psite embeddings decreases from 1.426 to 0.979. These results demonstrate that contrastive learning effectively enhances cross-view alignment and improves representation consistency.

We applied t-SNE (Van der Maaten and Hinton 2008) to visualize the feature space of CDVGL-PDA by projecting fused psite-disease representations into two dimensions. As shown in Fig. 4d, red and blue points denote positive and negative PDAs, respectively, with distributions shown before and after training. After training, positive and negative samples exhibit clearer separation in the projected space. This improvement is supported by quantitative metrics: the silhouette coefficient increases from 0.0458 to 0.2069, while the Davies-Bouldin index decreases from 4.6446 to 1.8804, indicating enhanced cluster compactness and inter-class separability. The relatively low absolute SC values are expected due to the inherent heterogeneity of PDAs, which involve diverse psite-disease combinations rather than forming compact clusters. Overall, these results suggest that CDVGL-PDA learns more discriminative representations, consistent with its improved predictive performance.

### 2.7 Ablation study

To evaluate the contribution of each core module, we conducted ablation experiments by: (i) removing the GRU and using BLOSUM62-encoded sequences directly, (ii) removing RGAT with its view-specific attention, (iii) removing HGT with its view-specific attention, and (iv) removing the contrastive learning constraint. As shown in Fig. 5a, removing the GRU resulted in the largest performance drop, with AUC and AUPR decreasing by 2.9% and 3.7%, respectively, highlighting the importance of sequence modeling. Removing RGAT, HGT, or contrastive learning also degraded performance, confirming their roles in capturing graph structure and enforcing multi-view consistency. The impact of removing RGAT exceeded that of HGT, consistent with its higher attention weight (60.83%), indicating a stronger contribution during information fusion. Overall, all modules jointly enhance the modeling of PDAs. Similar trends were observed under other low-similarity settings (SI Table S8), where the full model consistently achieved the best performance, and removing GRU, contrastive learning, or graph modules led to performance declines, particularly in AUPR. These findings demonstrate that module contributions remain consistent across both low-similarity and imbalanced scenarios.

**Figure 5:**
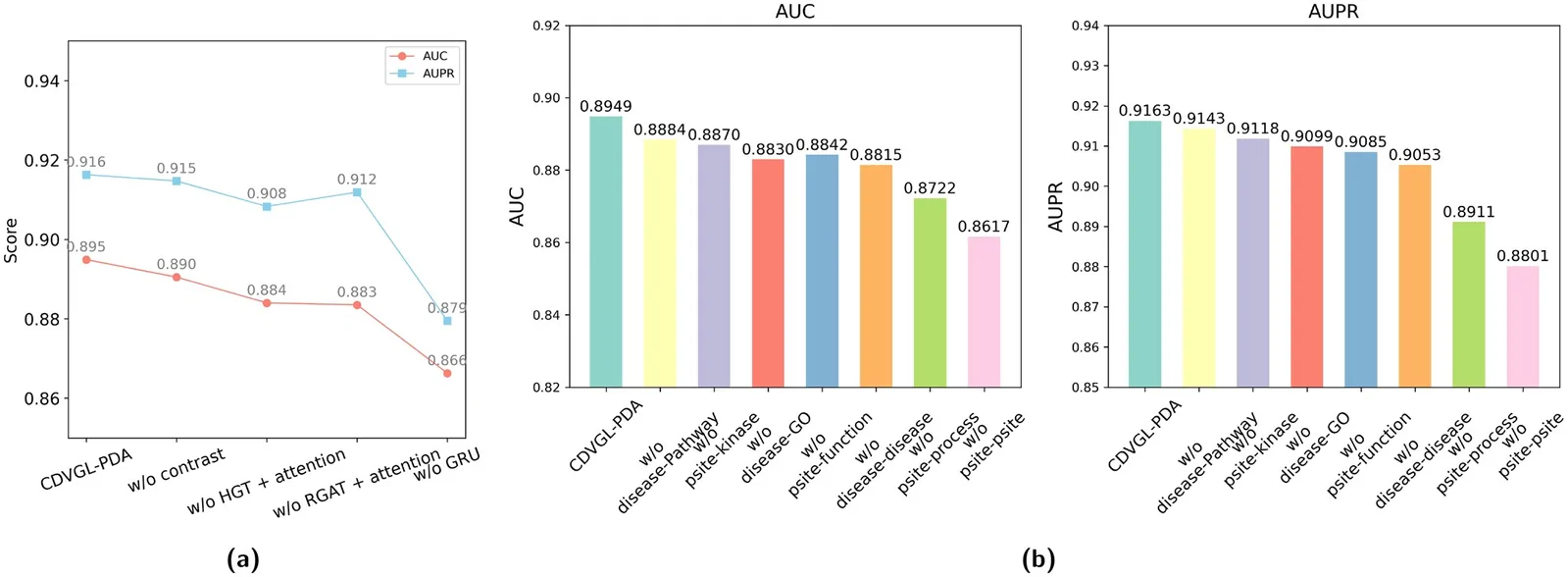
The ablation studies of CDVGL-PDA. (a) AUC and AUPR comparison of CDVGL-PDA under different module ablations. (b) AUC and AUPR comparison of CDVGL-PDA under different subnetwork ablations.

We further evaluated the contribution of different subnetworks by masking edges related to diseases or psites and assessing performance changes. As shown in Fig. 5b, masking the psite–psite edges caused the largest performance decline (AUC decreased by 3.32% and AUPR by 3.62%), highlighting their critical role. Masking other subnetworks also reduced performance, indicating that the model effectively integrates multi-source biological information. Overall, these results demonstrate the advantage of leveraging heterogeneous network structure and multi-modal information for improved predictive performance.

## 3 Materials and methods

### 3.1 PDAs data collection

PDAs were curated from PhosphoSitePlus (Hornbeck *et al.* 2015) (PSP) and PTMD2.0 (Huang *et al.* 2025), followed by integration and deduplication. Disease terms were standardized to Medical Subject Headings (MeSH). When no exact match was available, terms were mapped to appropriate higher-level MeSH categories. After preprocessing, 4232 confirmed PDA pairs were obtained, involving 2322 psites and 293 diseases. To reduce potential false negatives, we constructed a biologically constrained candidate negative pool (MeSH-constrained pool) based on second-level MeSH disease categories, which reduces the likelihood of sampling negative pairs that are semantically similar to known positive associations (details in SI Text S9).

To characterize dataset representativeness, we analysed the distribution of psites across diseases, proteins, and the MeSH hierarchy. Neoplasm-related diseases account for a large proportion, while non-neoplastic diseases remain well represented (details in SI Text S10 and SI Tables S9 and S10). Psites are broadly distributed across proteins without dominance by a few hubs (SI Text S11). In addition, analysis of the MeSH hierarchy reveals diverse association patterns, including parent–child relationships, intra-category sharing, and cross-category connections (SI Text S12 and SI Fig. S6).

### 3.2 Dataset construction

PDA prediction was formulated as a binary classification task. A balanced benchmark (Balance-1to1) was first constructed using all 4232 confirmed PDAs as positive samples and an equal number of randomly selected negatives from the MeSH-constrained pool. To evaluate robustness under class imbalance, two additional benchmarks were generated: Imbalance-1to5 and Imbalance-1toall. In both settings, all confirmed PDAs were retained as positives, while negatives were either randomly sampled at five times the positives (1:5) or fully included from the MeSH-constrained pool (1: all). These two benchmarks are designed to better reflect the real-world distribution of PDAs, where known positive associations are vastly outnumbered by non-associated pairs.

To assess model generalizability, we constructed three low-similarity evaluation benchmarks, namely protein-level split, psite-level split, and pair-level split, which are designed to evaluate performance under progressively stricter similarity constraints at the protein, psite, and pair levels, respectively. Detailed benchmark construction is provided in SI Text S13 and SI Table S11. Sensitivity analyses of different similarity threshold settings are presented in SI Text S14.

### 3.3 Baseline methods and evaluation metrics

To ensure the comparability of our experiments, we selected a set of reasonable baseline methods to comprehensively evaluate the performance of the proposed method: **MSF-CNN** (Deng *et al.* 2024) is a state-of-the-art PDA prediction method that integrates multiple similarity networks with Random Walk with Restart to capture topological information. **Heterogeneous graph representation learning** methods, including HAN (Wang *et al.* 2019), HGT (Hu *et al.* 2020), RGAT (Busbridge *et al.* 2019), GNN-FiLM (Brockschmidt 2020), RGCN (Schlichtkrull *et al.* 2018), and metapath2vec (Dong *et al.* 2017), exploit node features and network topology to capture high-order interactions in heterogeneous biological networks. **AutoML** approaches, such as TabPFN (Hollmann *et al.* 2025) and AutoGluon (Erickson *et al.* 2020), serve as modern baselines for tabular biological data. A detailed introduction to all baseline methods, including model descriptions, input features, and computational costs, is provided in SI Text S15 and SI Tables S12-15.

We use the area under the receiver operating characteristic curve (AUC), the area under the precision-recall curve (AUPR), and the F1 score as primary evaluation metrics. AUC measures the overall performance of the classifier but may be misleading for extremely imbalanced data, while AUPR emphasizes the prediction of minority classes and is more suitable for evaluating imbalanced datasets. The F1 score provides a balance between precision and recall, capturing the classifier’s effectiveness on positive samples.

### 3.4 Heterogeneous graph construction

To model the complex biological context underlying PDAs, we constructed a task-specific heterogeneous graph by integrating data from PSP, PTMD2.0, CTD (Davis *et al.* 2025), and UniProt (The UniProt Consortium 2025), as existing public biomedical knowledge graphs generally lack sufficient psite-level information for PDA prediction. The heterogeneous graph is defined as G=(V,E), where *V* and *E* denote the sets of nodes and edges, respectively. Nodes and edges are further categorized into types: *A* is the set of node types and *R* is the set of edge types. By definition, a graph is heterogeneous if it contains multiple node or edge types (|A|+|R|>2). The node types *A* include: psite, protein, pathway, molecular function, kinase, biological process, disease, gene, and GO. The edge types *R* include: molecular function–psite, kinase–psite, biological process–psite, protein–psite, psite–psite, disease–disease, gene–disease, GO–disease, pathway–disease, and gene–protein. The graph contains 15 937 nodes and 207 064 edges (Table 1). Detailed node and edge information is provided in SI Tables S26 and S27, and a representative subgraph is shown in SI Fig. S1. Further details of heterogeneous graph construction, including data sources, edge generation, and node feature construction, are provided in SI Text S16. Ablation analyses of psite encoding strategies are presented in SI Text S17.

### 3.5 Phosphorylation site encoder

We extract a sequence fragment of length L=71 centered at the psite, $S=[a_{0},\ldots ,a_{70}]$, and encode each residue using the 21-dimensional BLOSUM62 vector, yielding $X\in R^{B\times L\times 21}$. A learnable positional embedding $P\in R^{L\times 21}$ is added, $\tilde{X}=X+P$, followed by a linear projection to hidden dimension H=128. To capture local context, the sequence is split at the central residue (included in both segments) into left and right fragments:


$$H_{\text{left}}=H_{0}[:,:c,:],\;H_{\text{right}}=H_{0}[:,c-1:,:]$$

which are fed into bidirectional GRUs. Temporal pooling produces segment-level vectors $v_{\text{left}}$ and $v_{\text{right}}$, which are concatenated and linearly transformed to obtain the final psite representation $v_{\text{psite}}$.

### 3.6 Dual-view heterogeneous graph encoders

To capture diverse aspects of heterogeneous biological interactions, we employ two graph encoders: Relational Graph Attention Network (RGAT) and Heterogeneous Graph Transformer (HGT).

The RGAT module extends the Graph Attention Network (Veličković *et al.* 2017) to heterogeneous settings by maintaining independent attention mechanisms for each relation type r∈R. It focuses on capturing fine-grained local interaction patterns by adaptively weighting neighboring nodes within each relation. For a target node *t* of type τ(t), the hidden representation $h_{t}^{\left(l\right)}$ at the *l*-th layer is updated by aggregating messages from neighbors $s\in N_{t}^{r}$ across all heads i∈{1,…,H}. To ensure stable convergence across diverse interaction types, we employ a multi-layer architecture where the outputs of different relations are averaged:


(1) $$h_{t}^{\left(l\right)}={\text{Mean}}_{r\in R}(\overset{H}{\underset{i=1}{⊕}}\sum_{s\in N_{t}^{r}}\alpha_{st}^{(i),r}W^{(i),r}h_{s}^{\left(l-1\right)}),$$

where ⊕ represents the concatenation operation, $W^{(i),r}$ denotes the learnable weight matrix for the *i*-th head of relation *r*, and $h_{s}^{\left(l-1\right)}$ denotes the features of source neighbors from the preceding layer. The attention coefficients $\alpha_{st}^{(i),r}$ are computed locally within each relation, allowing the model to distinguish the importance of neighbors based on specific interaction types.

The HGT module explicitly models meta-relations 〈τ(s),ϕ(e),τ(t)〉 to capture high-level semantic dependencies (Hu *et al.* 2020). It captures global relational semantics by modeling interactions across node and edge types in a unified manner. For each meta-relation, type-specific projections generate queries, keys, and values, modulated by relation-specific tensors. Neighbor messages are aggregated via multi-head attention, followed by an adaptive gated skip connection to update the target node representation:


(2) $$h_{t}^{\left(l\right)}=\text{Norm}(g_{\tau (t)}\cdot {\tilde{h}}_{t}^{\left(l\right)}+(1-g_{\tau (t)})\cdot W_{\tau (t)}^{o}h_{t}^{\left(l-1\right)}),$$

where $g_{\tau (t)}=\sigma (\beta_{\tau (t)})$ is a learnable gating factor for node type τ(t). The term ${\tilde{h}}_{t}^{\left(l\right)}=\text{Aggregate}(\sum \alpha_{s,t}^{r}v_{s,t}^{r})$ represents the aggregated message from all neighbors under their respective relations. $h_{t}^{\left(l-1\right)}$ here refers to the target node’s own representation from the previous layer, facilitating a residual-like identity mapping for feature preservation. The projection $W_{\tau (t)}^{o}$ ensures feature alignment with the target dimension, effectively mitigating over-smoothing in deep heterogeneous networks.

### 3.7 Cross-view contrastive learning

To align node representations across different graph architectures and mitigate view-specific noise, we employ a cross-view contrastive learning mechanism based on the InfoNCE loss (He *et al.* 2020). This component enforces consistency between embeddings generated by the RGAT and HGT encoders. The raw node embeddings from both encoders are first projected into a shared latent space via a multi-layer perceptron.

For each node type k∈{disease, psite}, we define a symmetric InfoNCE loss. The forward contrastive loss, treating RGAT embeddings ($z_{i}^{\text{RGAT}}$) as anchors and the corresponding HGT embeddings ($z_{i}^{\text{HGT}}$) as positive pairs within a batch of size $N_{k}$, is formulated as:


(3) $$L_{k}^{\text{RGAT}\to \text{HGT}}=-\frac{1}{N_{k}}\sum_{i=1}^{N_{k}}\;\text{log}\;\frac{\;\text{exp}\;\left(\text{sim}(z_{i}^{\text{RGAT}},z_{i}^{\text{HGT}})/\tau \right)}{\sum_{j=1}^{N_{k}}\;\text{exp}\;\left(\text{sim}(z_{i}^{\text{RGAT}},z_{j}^{\text{HGT}})/\tau \right)}$$

where τ is the temperature parameter and sim(·,·) denotes cosine similarity. A symmetrical backward loss $L_{k}^{\text{HGT}\to \text{RGAT}}$ is similarly computed by alternating the anchor view.

The final contrastive objective for each node type is the average of these bidirectional losses:


(4) $$L_{k}=\frac{1}{2}\left(L_{k}^{\text{RGAT}\to \text{HGT}}+L_{k}^{\text{HGT}\to \text{RGAT}}\right)$$

### 3.8 Attention-weighted dual-view fusion

To adaptively integrate the complementary information from the RGAT and HGT encoders, we employ a semantic-level attention mechanism. For each node type k∈{disease, psite}, let $h_{k}^{\text{RGAT}}$ and $h_{k}^{\text{HGT}}$ denote the respective view-specific embeddings. We first compute the importance weight $\beta_{k}^{\left(i\right)}$ for each view i∈{RGAT, HGT} as follows:


(5) $$\beta_{k}^{\left(i\right)}=\frac{\;\text{exp}(a^{⊤}\text{tanh}(Wh_{k}^{\left(i\right)}+b))}{\sum_{j\in \{\text{RGAT},\;\text{HGT}\}}\;\text{exp}\;(a^{⊤}\text{tanh}(Wh_{k}^{\left(j\right)}+b))}$$

where a, W, and b are learnable attention parameters. The final fused representation $z_{k}$ is obtained by a weighted combination of the dual-view embeddings:


(6) $$z_{k}=\sum_{i\in \{\text{RGAT},\;\text{HGT}\}}\beta_{k}^{\left(i\right)}h_{k}^{\left(i\right)}$$

This approach allows the model to dynamically prioritize the most informative view for each node type, thereby enhancing the expressiveness of the final embeddings for downstream association prediction.

### 3.9 Association prediction with multi-task optimization

To predict PDAs, we adopt the ComplEx scoring function (Trouillon *et al.* 2016), which models asymmetric relations in a complex embedding space. Let $z_{p},z_{d}\in R^{d}$ denote the final fused embeddings of a psite node *p* and a disease node *d*. These embeddings are projected into the complex space $C^{d/2}$ by splitting them into real and imaginary parts: $z=z^{\text{re}}+iz^{\text{im}},z^{\text{re}},z^{\text{im}}\in R^{d/2}.$ Based on this representation, the association score for a pair (p,d) is defined as:


(7) $$\begin{array}{cc} f(z_{p},z_{d})=\text{Re}(\langle r,z_{p},{\bar{z}}_{d}\rangle ) & \\ =\sum_{l=1}^{d/2}(r_{l}^{\text{re}}z_{p,l}^{\text{re}}z_{d,l}^{\text{re}}+r_{l}^{\text{re}}z_{p,l}^{\text{im}}z_{d,l}^{\text{im}}\\ +r_{l}^{\text{im}}z_{p,l}^{\text{re}}z_{d,l}^{\text{im}}-r_{l}^{\text{im}}z_{p,l}^{\text{im}}z_{d,l}^{\text{re}}) & \end{array}$$

where ${\bar{z}}_{d}$ denotes the complex conjugate of the disease embedding, and *l* indexes the latent dimensions in the complex plane.

We train the model end-to-end using a multi-task objective. The association score is treated as a logit and optimized via binary cross-entropy: $L_{\text{BCE}}=-(y\;\text{log}\;\sigma (f)+(1-y)\;\text{log}(1-\sigma (f)))$, where y∈{0,1} denotes the ground-truth label for the PDA pair. To improve representation robustness, contrastive losses are applied to both psite and disease nodes. The overall objective is:


(8) $$L_{\text{total}}=\lambda_{1}L_{\text{psite}}+\lambda_{2}L_{\text{disease}}+\lambda_{3}L_{\text{BCE}},$$

where $\lambda_{1},\lambda_{2},\lambda_{3}$ control the contributions of each task.

## 4 Conclusion

In this study, we propose a contrastive dual-view graph learning framework, CDVGL-PDA, for predicting PDAs. As summarized in Table S16, our framework incorporates four core innovations to improve PDA prediction performance. We construct a heterogeneous graph to model phosphorylation-centric networks and initialize node features using modality-specific encodings. A dual-view encoding strategy with RGAT and HGT is then applied to learn complementary representations, which are aligned via contrastive learning and integrated through attention-based fusion. The resulting unified embeddings are used for PDA prediction via a scoring function. Comprehensive experiments, including benchmark comparisons, ablation studies, biological validation analyses, and interpretability analyses, demonstrate the effectiveness and robustness of CDVGL-PDA. In addition, case studies show that several high-confidence predicted PDAs are supported by published literature evidence. This finding indicates that CDVGL-PDA has the potential to uncover previously unrecognized PDAs and provide valuable candidates for subsequent biological investigation.

CDVGL-PDA is built upon a heterogeneous graph, enabling the effective integration of diverse biological and biomedical information and enhancing the representation of psites and diseases. However, the method is inherently limited by the transductive nature of heterogeneous graph learning: psites and diseases to be predicted must be present in the graph, preventing direct prediction for unseen nodes. In future work, we aim to incorporate inductive graph learning to dynamically integrate unseen nodes into the framework, enabling inference of associations not explicitly present in the original graph. In addition, we plan to enhance node feature representations to further improve predictive performance.

## Supplementary Material

btag690_Supplementary_Data

## Data Availability

The source code and datasets for CDVGL-PDA are publicly available in the GitHub repository at https://github.com/ljquanlab/CDVGL-PDA/.

## References

[btag690-B1] Brockschmidt M. Gnn-film: graph neural networks with feature-wise linear modulation. In *International Conference on Machine Learning*. PMLR, 2020, 1144–1152.

[btag690-B2] Busbridge D, Sherburn D, Cavallo P et al Relational graph attention networks. arXiv preprint, arXiv:1904.05811, 2019, preprint: not peer reviewed.

[btag690-B3] Chakraborty P, Ibáñez de Opakua A, Purslow JA et al Gsk3β phosphorylation catalyzes the aggregation of tau into Alzheimer’s disease-like filaments. Proc Natl Acad Sci 2024;121:e2414176121. 10.1073/pnas.2414176121 PMC11670061 39693350

[btag690-B4] Chen Y , Zhou S, Wan K et al Riok1 mediates p53 degradation and radioresistance in colorectal cancer through phosphorylation of g3bp2. Oncogene 2022;41:3433–44. 10.1038/s41388-022-02352-4 35589951

[btag690-B5] Davis AP , Wiegers TC, Sciaky D et al Comparative toxicogenomics database’s 20th anniversary: update 2025. Nucleic Acids Res 2025;53:D1328–34. 10.1093/nar/gkae883 PMC11701581 39385618

[btag690-B6] Deng Q , Zhang J, Liu J et al Identifying protein phosphorylation site-disease associations based on multi-similarity fusion and negative sample selection by convolutional neural network. Interdiscip Sci 2024;16:649–64. 10.1007/s12539-024-00615-0 38457108

[btag690-B7] Dong Y, Chawla NV, Swami A. metapath2vec: scalable representation learning for heterogeneous networks. In: *Proceedings of the 23rd ACM SIGKDD International Conference on Knowledge Discovery and Data Mining*. 2017, 135–144.

[btag690-B8] Dubois-Deruy E , Belliard A, Mulder P et al Interplay between troponin t phosphorylation and o-n-acetylglucosaminylation in ischaemic heart failure. Cardiovasc Res 2015;107:56–65. 10.1093/cvr/cvv136 25916824

[btag690-B9] Erickson N , Mueller J, Shirkov A et al Autogluon-tabular: Robust and accurate automl for structured data. arXiv preprint, arXiv:2003.06505, 2020, preprint: not peer reviewed.

[btag690-B10] He J , Wu Y, Pu X et al A transfer-learning-based deep convolutional neural network for predicting leukemia-related phosphorylation sites from protein primary sequences. Int J Mol Sci 2022;23:1741. 10.3390/ijms23031741 PMC8915183 35163663

[btag690-B11] He K, Fan H, Wu Y et al Momentum contrast for unsupervised visual representation learning. In: *Proceedings of the IEEE/CVF Conference on Computer Vision and Pattern Recognition*. 2020, 9729–9738.

[btag690-B12] Hollmann N , Müller S, Purucker L et al Accurate predictions on small data with a tabular foundation model. Nature 2025;637:319–26. 10.1038/s41586-024-08328-6 PMC11711098 39780007

[btag690-B13] Hornbeck PV , Zhang B, Murray B et al Phosphositeplus, 2014: mutations, ptms and recalibrations. Nucleic Acids Res 2015;43:D512–D520. 10.1093/nar/gku1267 PMC4383998 25514926

[btag690-B14] Hossain MS , Karuniawati H, Jairoun AA et al Colorectal cancer: a review of carcinogenesis, global epidemiology, current challenges, risk factors, preventive and treatment strategies. Cancers (Basel) 2022;14:1732. 10.3390/cancers14071732 PMC8996939 35406504

[btag690-B15] Hu Z, Dong Y, Wang K, Sun Y et al Heterogeneous graph transformer. In: *Proceedings of the Web Conference 2020*. 2020, 2704–2710.

[btag690-B16] Huang X , Feng Z, Liu D et al Ptmd 2.0: an updated database of disease-associated post-translational modifications. Nucleic Acids Res 2025;53:D554–D563. 10.1093/nar/gkae850 PMC11701619 39329270

[btag690-B17] Knopman DS , Amieva H, Petersen RC et al Alzheimer disease. Nat Rev Dis Primers 2021;7:33. 10.1038/s41572-021-00269-y PMC8574196 33986301

[btag690-B18] Leutert M , Entwisle SW, Villén J et al Decoding post-translational modification crosstalk with proteomics. Mol Cell Proteomics 2021;20:100129. 10.1016/j.mcpro.2021.100129 PMC8430371 34339852

[btag690-B19] Mann M , Ong SE, Grønborg M et al Analysis of protein phosphorylation using mass spectrometry: deciphering the phosphoproteome. Trends Biotechnol 2002;20:261–8. 10.1016/s0167-7799(02)01944-3 12007495

[btag690-B20] McInnes L , Healy J, Melville J. Umap: Uniform manifold approximation and projection for dimension reduction. arXiv preprint, arXiv:1802.03426, 2018, preprint: not peer reviewed.

[btag690-B21] Olsen JV , Blagoev B, Gnad F et al Global, in vivo, and site-specific phosphorylation dynamics in signaling networks. Cell 2006;127:635–48. 10.1016/j.cell.2006.09.026 17081983

[btag690-B22] Pham NT , Phan LT, Seo J et al Advancing the accuracy of sars-cov-2 phosphorylation site detection via meta-learning approach. Brief Bioinform 2024;25:bbad433. 10.1093/bib/bbad433 PMC10753650 38058187

[btag690-B23] Sabir MJ , Kamli MR, Atef A et al Computational prediction of phosphorylation sites of sars-cov-2 infection using feature fusion and optimization strategies. Methods 2024;229:1–8. 10.1016/j.ymeth.2024.04.021 38768932

[btag690-B24] Sato C , Mallipeddi N, Ghoshal N et al Mapt r406w increases tau t217 phosphorylation in absence of amyloid pathology. Ann Clin Transl Neurol 2021;8:1817–30. 10.1002/acn3.51435 PMC8419397 34342183

[btag690-B25] Schlichtkrull M, Kipf TN, Bloem P et al Modeling relational data with graph convolutional networks. In: *European Semantic Web Conference*. Springer, 2018, 593–607.

[btag690-B26] Song W , Xu L, Han C et al Drug–target interaction predictions with multi-view similarity network fusion strategy and deep interactive attention mechanism. Bioinformatics 2024;40:btae346. 10.1093/bioinformatics/btae346 PMC11164831 38837345

[btag690-B27] Trouillon T, Welbl J, Riedel S et al Complex embeddings for simple link prediction. In: *International Conference on Machine Learning*. PMLR, 2016, 2071–2080.

[btag690-B28] The UniProt Consortium. Uniprot: the universal protein knowledgebase in 2025. Nucleic Acids Res 2025;53:D609–D617. 10.1093/nar/gkae1010 PMC11701636 39552041

[btag690-B29] Van der Maaten L , Hinton G. Visualizing data using t-sne. J Mach Learn Res 2008;9:2579–605.

[btag690-B30] Veličković P, Cucurull G, Casanova A et al Graph attention networks. arXiv preprint, arXiv:1710.10903, 2017, preprint: not peer reviewed.

[btag690-B31] Wang X, Ji H, Shi C et al Heterogeneous graph attention network. In: *The World Wide Web Conference*. 2019, 2022–2032.

[btag690-B32] Xiao L , Harrison DG. Inflammation in hypertension. Can J Cardiol 2020;36:635–47. 10.1016/j.cjca.2020.01.013 PMC7733126 32389337

[btag690-B33] Xie J , Dong R, Zhu J et al Mmfuncphos: a multi-modal learning framework for identifying functional phosphorylation sites and their regulatory types. Adv Sci 2025;12:2410981. 10.1002/advs.202410981 PMC11884596 39804866

[btag690-B34] Xu X , Wang M. Inferring disease associated phosphorylation sites via random walk on multi-layer heterogeneous network. IEEE/ACM Trans Comput Biol Bioinform 2015;13:836–44. 10.1109/TCBB.2015.2498548 26584500

[btag690-B35] Xu X , Li A, Wang M et al Prediction of human disease-associated phosphorylation sites with combined feature selection approach and support vector machine. IET Syst Biol 2015;9:155–63. 10.1049/iet-syb.2014.0051 PMC8687269 26243832

[btag690-B36] Ye H , Han Y, Li P et al The role of post-translational modifications on the structure and function of tau protein. J Mol Neurosci 2022;72:1557–71. 10.1007/s12031-022-02002-0 35325356

[btag690-B37] Zhang B , Quan L, Zhang Z et al Mvcl-dti: predicting drug–target interactions using a multiview contrastive learning model on a heterogeneous graph. J Chem Inf Model 2025;65:1009–26. 10.1021/acs.jcim.4c02073 39812134

[btag690-B38] Zhang D , Wong LL, Koay ESC et al Phosphorylation of ser78 of hsp27 correlated with her-2/neu status and lymph node positivity in breast cancer. Mol Cancer 2007;6:52. 10.1186/1476-4598-6-52 PMC1976621 17697330

[btag690-B39] Zhang G , Zhang C, Cai M et al Funcphos-str: an integrated deep neural network for functional phosphosite prediction based on alphafold protein structure and dynamics. Int J Biol Macromol 2024;266:131180. 10.1016/j.ijbiomac.2024.131180 38552697

