# Supplementary material for: Integrating multi-modal biological knowledge via contrastive dual-view graph learning for phosphorylation site-disease association prediction
Source: Bioinformatics. 2026 Sep 18;42(10):btag690. doi: 10.1093/bioinformatics/btag690 (PMC13633665; doi:10.1093/bioinformatics/btag690)
Supplement: Supplementary file 1 [file btag690_supplementary_data.zip › Supplement.pdf]

Integrating Multi–Modal Biological Knowledge via Contrastive Dual–View Graph Learning for Phosphorylation Site–Disease Association Prediction

Supplementary Figure:

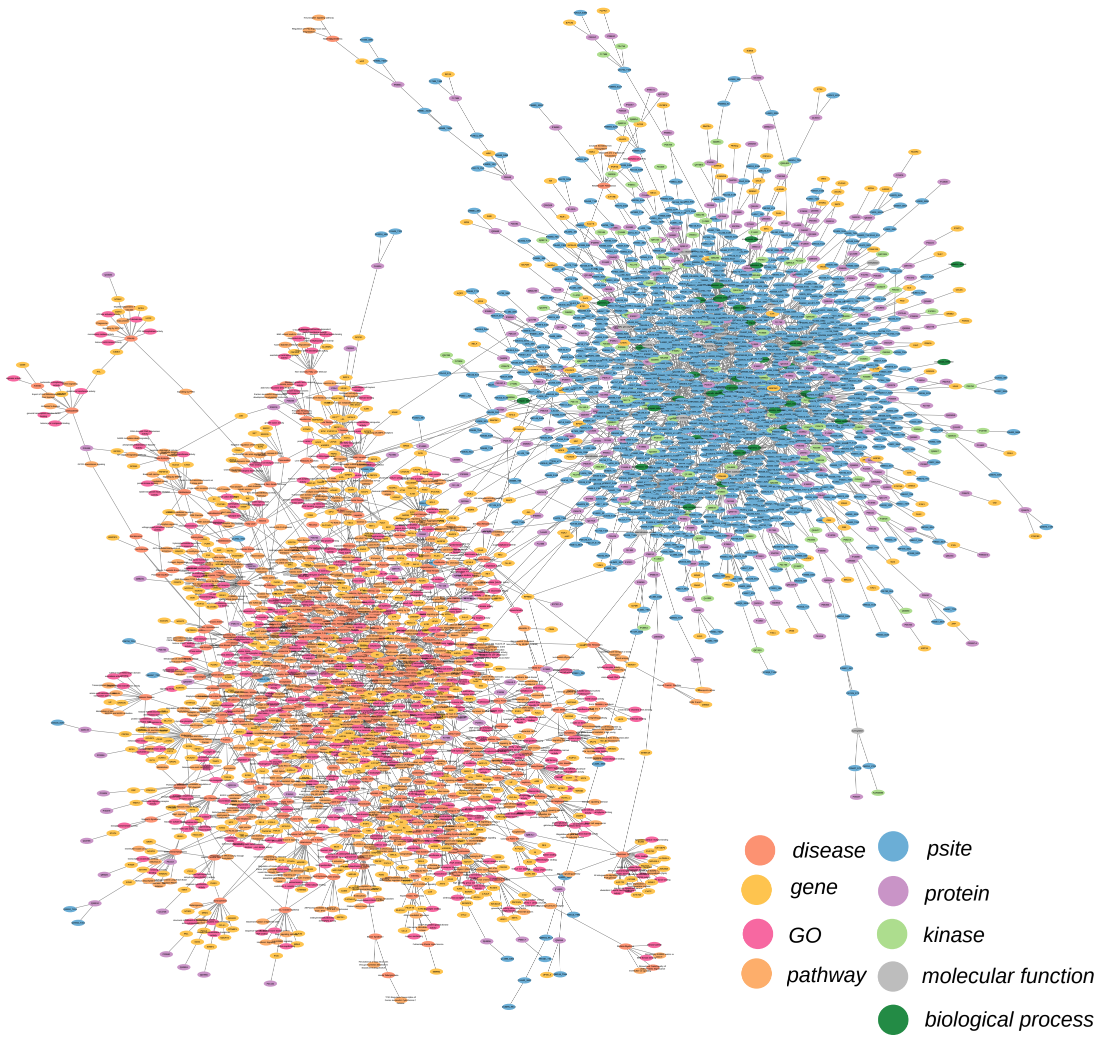

Figure S1. A representative heterogeneous subgraph. The subgraph was obtained by randomly sampling 500 edges per edge type from the full graph (15,937 nodes, 207,064 edges) and removing some isolated edges after fusion.

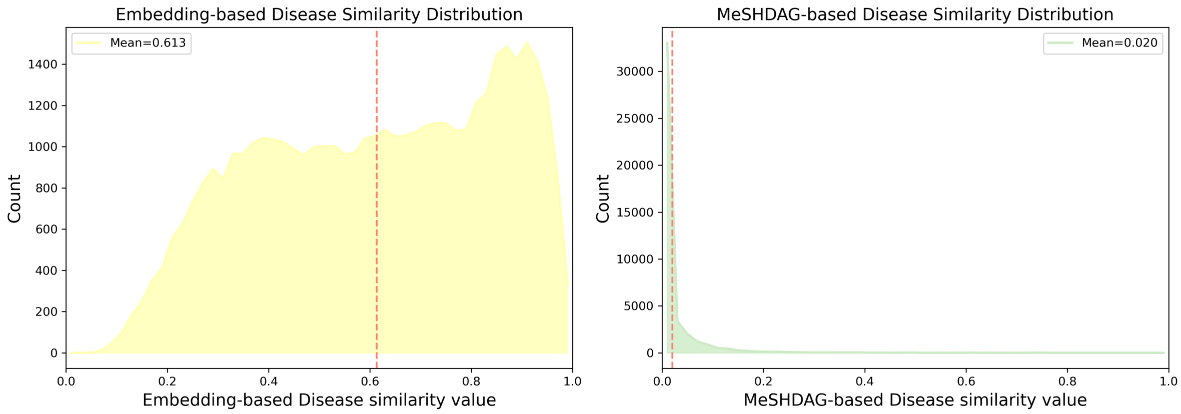

Figure S2. The distribution of embedding-based disease similarity and MeSHDAG-based disease similarity.

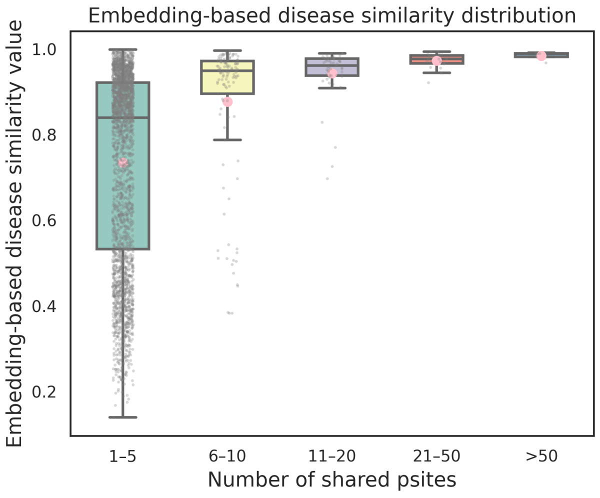

(a)

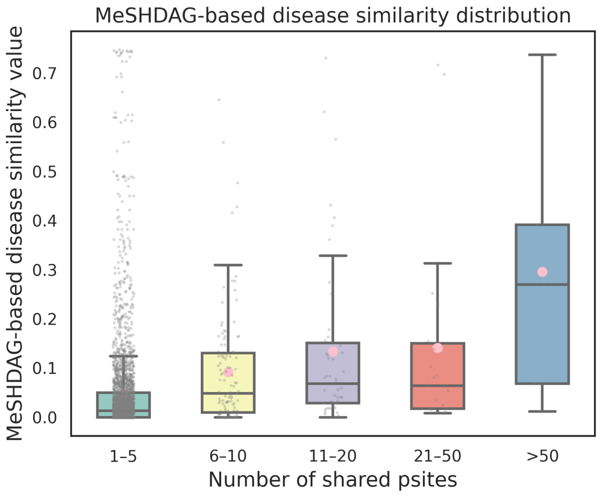

(b)

Figure S3. (a) Positive correlation between the number of shared psites and embedding-based disease similarity. Boxplots show median (central line), mean (circle marker), interquartile range (boxes), and individual pairs (gray dots with jitter). (b) Positive correlation between the number of shared psites and MeSHDAG-based disease similarity.

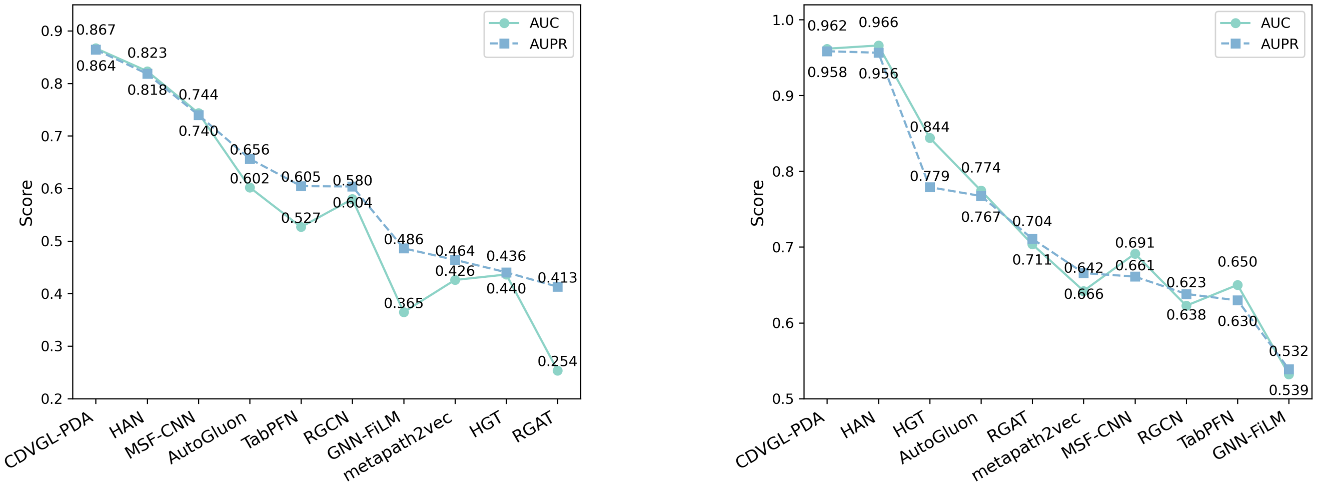

(a) **(b)**

Figure S4. Performance of CDVGL-PDA in predicting disease-specific psites. (a) AUC and AUPR comparison of CDVGL-PDA and baseline methods in predicting psite-Alzheimer Disease associations. (b) AUC and AUPR comparison of CDVGL-PDA and baseline methods in predicting psite-Colorectal Neoplasms associations.

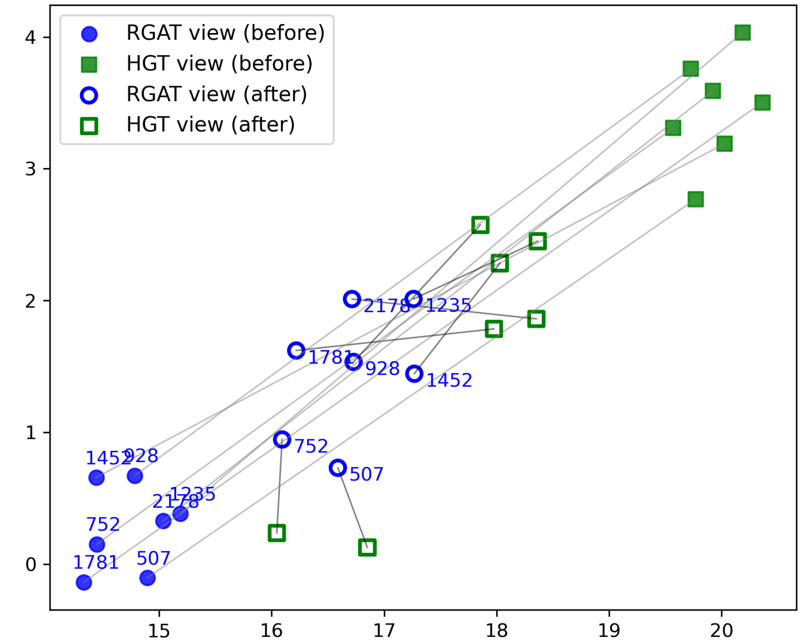

Figure S5. Visualization of psite embeddings associated with Lung Neoplasms before and after contrastive learning. Psite embeddings are projected into a two-dimensional space using UMAP. Solid points represent embeddings before contrastive learning, whereas hollow points represent embeddings after contrastive learning. Blue circles denote the RGAT view, and green squares denote the HGT view. For the same psite, embeddings from the two views are connected by a line, and the numbers labeled in the RGAT view correspond to psite indices.

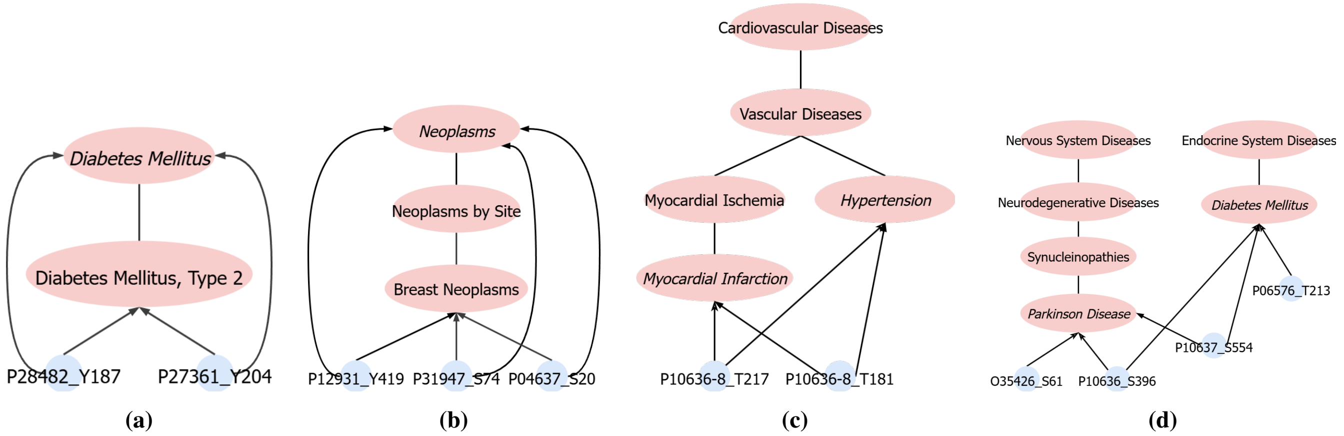

Figure S6. Representative examples illustrating the distribution of psites in the context of the MeSH disease hierarchy. Red ellipses represent diseases, blue circles represent psites. Solid arrows indicate confirmed PDAs, while dashed arrows indicate predicted PDAs. (a) Distribution of psites from specific disease subtypes to their parent diseases. (b) Hierarchical spread of psites from neoplasm subtypes to broader neoplasm categories. (c) Shared psites across diseases within the Vascular Diseases category. (d) Cross-system distribution of psites highlighting potential comorbidity.

Supplementary Tables:

Table S1. Performance comparison of CDVGL-PDA and other baseline methods on the Balanced benchmark using 10-fold cross-validation.

Method	AUPR	AUC	F1	ACC	Precision	Recall	MCC
metapath2vec	0.8611 ± 0.0333	0.8488 ± 0.0312	0.7713 ± 0.0204	0.7747 ± 0.0321	0.7917 ± 0.0563	0.7559 ± 0.0294	0.5529 ± 0.0625
RGCN	0.8805 ± 0.0171	0.8761 ± 0.0192	0.8111 ± 0.0201	0.8087 ± 0.0235	0.8052 ± 0.0430	0.8204 ± 0.0391	0.6201 ± 0.0455
GNN-FiLM	0.8874 ± 0.0104	0.8849 ± 0.0098	0.8212 ± 0.0085	0.8241 ± 0.0078	0.8351 ± 0.0129	0.8081 ± 0.0181	0.6488 ± 0.0155
RGAT	0.8973 ± 0.0117	0.8957 ± 0.0109	0.7626 ± 0.0456	0.6963 ± 0.0908	0.6408 ± 0.0711	**0.9530 ± 0.0293**	0.4492 ± 0.1588
MSF-CNN	0.8978 ± 0.0096	0.8981 ± 0.0064	0.8382 ± 0.0096	0.8404 ± 0.0081	0.8498 ± 0.0104	0.8273 ± 0.0202	0.6813 ± 0.0161
HGT	0.9067 ± 0.0171	0.9017 ± 0.0155	0.8308 ± 0.0206	0.8318 ± 0.0206	0.8377 ± 0.0370	0.8268 ± 0.0416	0.6658 ± 0.0400
TabPFN	0.9105 ± 0.0048	0.9050 ± 0.0065	0.8398 ± 0.0068	0.8438 ± 0.0065	0.8619 ± 0.0105	0.8190 ± 0.0121	0.6886 ± 0.0130
AutoGluon	0.9159 ± 0.0067	0.9085 ± 0.0088	0.8423 ± 0.0086	0.8452 ± 0.0088	0.8589 ± 0.0142	0.8266 ± 0.0118	0.6911 ± 0.0179
HAN	0.9464 ± 0.0073	0.9393 ± 0.0087	0.8664 ± 0.0168	0.8651 ± 0.0181	0.8595 ± 0.0275	0.8740 ± 0.0171	0.7308 ± 0.0361
CDVGL-PDA	**0.9562 ± 0.0059**	**0.9515 ± 0.0066**	**0.8892 ± 0.0121**	**0.8892 ± 0.0134**	**0.8909 ± 0.0289**	0.8885 ± 0.0166	**0.7791 ± 0.0274**

Table S2. Performance of CDVGL-PDA and other baseline methods on the Imbalance-1to5(an imbalanced benchmark with positive-to-negative ratio of 1:5) using 10-fold cross-validation.

Method	AUPR	AUC	F1	ACC	Precision	Recall	MCC
metapath2vec	0.5821 ± 0.1647	0.8137 ± 0.0899	0.4630 ± 0.1734	0.8055 ± 0.2135	0.6292 ± 0.2774	0.4369 ± 0.2265	0.4083 ± 0.2054
MSF-CNN	0.7541 ± 0.0217	0.9031 ± 0.0119	0.6788 ± 0.0254	0.9064 ± 0.0067	0.7934 ± 0.0297	0.5943 ± 0.0337	0.6348 ± 0.0274
RGCN	0.7619 ± 0.1037	0.9060 ± 0.0295	0.6552 ± 0.1605	0.9077 ± 0.0315	0.8264 ± 0.0857	0.5714 ± 0.1891	0.6297 ± 0.1437
RGAT	0.7786 ± 0.0432	0.9178 ± 0.0123	0.5720 ± 0.0708	0.7718 ± 0.0583	0.4305 ± 0.0955	**0.8838 ± 0.0391**	0.5027 ± 0.0776
TabPFN	0.8067 ± 0.0202	0.9197 ± 0.0090	0.7059 ± 0.0237	0.9162 ± 0.0063	0.8492 ± 0.0243	0.6042 ± 0.0247	0.6717 ± 0.0266
AutoGluon	0.8258 ± 0.0156	0.9247 ± 0.0076	0.7312 ± 0.0214	0.9212 ± 0.0060	0.8467 ± 0.0246	0.6437 ± 0.0226	0.6950 ± 0.0244
HGT	0.8664 ± 0.0158	0.9407 ± 0.0100	0.7974 ± 0.0160	0.9374 ± 0.0043	0.8665 ± 0.0274	0.7401 ± 0.0329	0.7646 ± 0.0170
GNN-FiLM	0.8729 ± 0.0095	0.9400 ± 0.0077	0.7980 ± 0.0155	0.9388 ± 0.0042	0.8877 ± 0.0235	0.7257 ± 0.0271	0.7681 ± 0.0169
HAN	0.8855 ± 0.0196	0.9508 ± 0.0094	0.8072 ± 0.0244	0.9393 ± 0.0079	0.8601 ± 0.0460	0.7632 ± 0.0403	0.7743 ± 0.0287
CDVGL-PDA	**0.9320 ± 0.0119**	**0.9695 ± 0.0063**	**0.8732 ± 0.0135**	**0.9593 ± 0.0040**	**0.9071 ± 0.0144**	0.8421 ± 0.0231	**0.8500 ± 0.0153**

Table S3. Performance of CDVGL-PDA and other baseline methods on the Imbalance-1toall(an imbalanced benchmark with positive-to-negative ratio of 1:all) using 10-fold cross-validation.

Method	AUPR	AUC	F1	ACC	Precision	Recall	MCC
metapath2vec	0.0774 ± 0.0543	0.7108 ± 0.1307	0.0020 ± 0.0061	0.8919 ± 0.2939	0.0010 ± 0.0031	0.1000 ± 0.3000	0.0000 ± 0.0000
AutoGluon	0.1994 ± 0.0483	0.8861 ± 0.0266	0.0501 ± 0.0862	0.9899 ± 0.0002	0.3133 ± 0.4078	0.0310 ± 0.0574	0.0822 ± 0.1098
HAN	0.2242 ± 0.0985	0.7374 ± 0.0782	0.0180 ± 0.0218	0.9899 ± 0.0001	0.6786 ± 0.4486	0.0092 ± 0.0112	0.0691 ± 0.0615
TabPFN	0.2736 ± 0.0626	0.8879 ± 0.0359	0.0136 ± 0.0291	0.9899 ± 0.0002	0.1500 ± 0.3202	0.0071 ± 0.0152	0.0323 ± 0.0695
GNN-FiLM	0.3556 ± 0.0240	0.8779 ± 0.0113	0.1864 ± 0.0269	0.9908 ± 0.0002	0.9012 ± 0.0346	0.1042 ± 0.0169	0.3039 ± 0.0266
MSF-CNN	0.3821 ± 0.0189	0.8991 ± 0.0125	0.2211 ± 0.0235	0.9909 ± 0.0001	0.8420 ± 0.0441	0.1276 ± 0.0155	0.3249 ± 0.0205
HGT	0.4825 ± 0.1745	0.8907 ± 0.0426	0.4132 ± 0.2493	0.9902 ± 0.0053	0.5591 ± 0.3951	0.3544 ± 0.1927	0.4310 ± 0.2641
RGCN	0.5827 ± 0.0689	0.8464 ± 0.0405	0.3663 ± 0.2741	0.9925 ± 0.0021	0.6992 ± 0.4577	0.2582 ± 0.2032	0.4142 ± 0.2913
RGAT	0.7910 ± 0.0227	0.9623 ± 0.0064	0.6807 ± 0.0301	0.9927 ± 0.0012	0.6228 ± 0.0664	**0.7597 ± 0.0416**	0.6824 ± 0.0269
CDVGL-PDA	**0.8593 ± 0.0183**	**0.9718 ± 0.0048**	**0.8505 ± 0.0158**	**0.9973 ± 0.0002**	**0.9910 ± 0.0061**	0.7453 ± 0.0261	**0.8581 ± 0.0138**

Table S4. Correlated Bayesian statistical comparison between CDVGL-PDA and the second-best method based on 10-fold cross-validation. Posterior probability represents $P(\text{CDVGL-PDA} > \text{the second-best method})$.

Metric	positive-to-negative ratios	Posterior probability	Paired t-test p-value
AUPR	1:1	0.9871	0.0059
	1:5	0.9998	0.0001
	1:all	0.9999	0.0001
AUC	1:1	0.9968	0.0012
	1:5	0.9997	0.0001
	1:all	0.9999	0.0001

Table S5. Performance comparison of different methods on pair-level, protein-level, and psite-level split benchmarks.

	Pair-level			Protein-level			Psite-level		
Method	AUPR	AUC	F1	AUPR	AUC	F1	AUPR	AUC	F1
metapath2vec	0.6135	0.6247	0.4265	0.8069	0.7938	0.6931	0.8235	0.7996	0.7441
RGAT	0.7131	0.6704	0.5352	0.8481	0.8394	0.7660	0.8817	0.8597	0.7294
GNN-FiLM	0.7141	0.6737	0.3876	0.8779	0.8582	0.7976	0.8849	0.8624	0.8031
HGT	0.7432	0.7008	0.4848	0.8658	0.8468	0.7751	0.8713	0.8445	0.7883
MSF-CNN	0.7511	0.7217	0.3715	0.8718	0.8572	0.7392	0.8442	0.8087	0.7098
TabPFN	0.7554	0.7135	0.3896	<u>0.8890</u>	0.8632	0.8016	<u>0.8972</u>	0.8715	0.8063
AutoGluon	0.7572	0.7262	0.3889	0.8811	<u>0.8646</u>	<u>0.8065</u>	0.8941	<u>0.8719</u>	**0.8210**
RGCN	0.7713	0.7051	0.5110	0.8468	0.8428	0.7280	0.8653	0.8579	0.7874
HAN	<u>0.7909</u>	<u>0.7531</u>	<u>0.6198</u>	0.7931	0.7830	0.6990	0.8814	0.8622	0.7733
CDVGL-PDA	**0.8458**	**0.8152**	**0.6368**	**0.8936**	**0.8947**	**0.8300**	**0.9163**	**0.8949**	<u>0.8119</u>

Table S6. Performance stratified by node degree on different benchmarks.

**Degree group**	**Protein-level split**			**Psite-level split**			**Pair-level split**		
	Number of pairs	Average degree	AUC	Number of pairs	Average degree	AUC	Number of pairs	Average degree	AUC
Low	132	105	0.7515	172	106	0.6169	212	59	0.7075
Medium	263	911	0.8896	342	887	0.8909	422	471	0.8357
High	131	2643	0.8533	172	2475	0.9057	212	1462	0.8285

Table S7. Number and proportion of disease–disease pairs with low MeSH similarity but high embedding similarity that share common neighbors in the heterogeneous network.

MeSH similarity value	Embedding similarity value	Total disease–disease pairs	Pairs with common neighbors	Proportion (%)
$0.01 \le$ value < 0.02	$0.7 \le$ value < 0.8	335	286	85.37
$0.005 \le$ value < 0.01	$0.8 \le$ value < 0.9	389	350	89.97
$0 \le$ value < 0.005	$0.9 \le$ value < 1	983	931	94.71

Table S8. Ablation study results under low-similarity settings.

Module	Psite-level split		Protein-level split		Pair-level split	
	AUC	AUPR	AUC	AUPR	AUC	AUPR
All(CDVGL-PDA)	0.8949	0.9163	0.8947	0.8936	0.8152	0.8458
w/o contrast	0.8905	0.9147	0.8919	0.8915	0.7947	0.8333
w/o HGT + attention	0.8840	0.9083	0.8930	0.8906	0.7987	0.8371
w/o RGAT + attention	0.8835	0.9119	0.8792	0.8644	0.7825	0.8234
w/o GRU	0.8663	0.8795	0.8727	0.8911	0.7631	0.7789

Table S9. Distribution of unique phosphorylation sites (psites) across the top 20 most frequent diseases.

Disease (MeSH)	Number of Unique Phosphorylation Sites
Breast Neoplasms	322
Carcinoma	255
Alzheimer Disease	231
Colorectal Neoplasms	175
Neoplasms	164
Prostatic Neoplasms	128
Carcinoma, Hepatocellular	115
Carcinoma, Non-Small-Cell Lung	115
Lung Neoplasms	106
Ovarian Neoplasms	102
Glioblastoma	96
Parkinson Disease	95
Diabetes Mellitus	90
Diabetes Mellitus, Type 2	80
Squamous Cell Carcinoma of Head and Neck	73
Neuroblastoma	69
Carcinoma, Pancreatic Ductal	63
Adenocarcinoma	60
Cardiomyopathies	58
Stomach Neoplasms	56

Table S10. Protein coverage of the top 20 proteins ranked by the number of unique psites.

Protein	Number of Unique Phosphorylation Sites
P10636-8	42
P10636	21
P10637	16
P46937	15
P10451	15
P10275	14
P07197	14
O95644	14
P03372	13
P19429	13
P12036	12
P42858	12
P35222	12
P06400	10
P00533	10
Q05397	9
P08047	9
P51608-2	8
P60484	8
P56945	8

Table S11. Statistics of the benchmarks on generalization benchmarks.

**Benchmark**	**Train**			**Test**		
	Positive pairs	Negative pairs	Unique psites	Positive pairs	Negative pairs	Unique psites
Protein-Level split	3969	3969	2141	263	263	181
Psite-Level split	3889	3889	2090	343	343	232
Pair-Level split	3809	3809	-	423	423	-

Table S12. Hyperparameter settings of some baseline methods.

Method	Hyperparameter	Value
HGT / RGCN / RGAT / GNN-FiLM / HAN	Learning rate	0.001
	Layers of graph neural networks	2
	Dimensions of hidden layers	512
	Weight decay	0.0005
	Number of neurons for first dense layer in MLP module	1024
	Number of neurons for second dense layer in MLP module	128
metapath2vec	min_count	3
	Number of workers	8
	Context window size	7
	Embedding dimensions	128
	Number of neurons for first dense layer in MLP module	256
	Number of neurons for second dense layer in MLP module	128

Table S13. Brief description of Baseline Methods

Model	Brief description
MSF-CNN [1]	MSF-CNN constructs multiple similarity networks for psites and diseases, applies the Random Walk with Restart algorithm to capture topological features, and performs dimensionality reduction using diffusion component analysis. The resulting features are fed into a CNN to identify potential PDAs.
TabPFN [2]	Tabular Prior-Data Fitted Network is a transformer-based model for tabular data that leverages prior-data fitting to approximate Bayesian inference, using an encoder-only architecture with in-context learning and attention mechanisms to capture label-feature relationships without positional encoding. It is directly used as a predictor for binary classification.
AutoGluon [3]	AutoGluon is an automated machine learning framework that constructs ensemble models by integrating diverse base learners, including gradient boosting methods (LightGBM, LightGBMXT, LightGBMLarge, XGBoost), bagging-based models (RandomForestGini, RandomForestEntr, ExtraTreesGini, ExtraTreesEntr), neural networks (NeuralNetTorch, NeuralNetFastAI), and CatBoost. Through automated model selection, hyperparameter optimization, and ensemble learning, it identifies optimal configurations for the given dataset and is employed for binary classification tasks.
HGT [4]	Heterogeneous Graph Transformer that constructs type-specific linear projections and employs multi-head, relation-aware attention to propagate messages across heterogeneous nodes and edges, with the resulting node embeddings fed into a multilayer perceptron (MLP) for prediction.
RGCN [5]	Relational Graph Convolutional Network that aggregates messages from heterogeneous neighbors using relation-specific weight matrices, producing node embeddings which are then processed by an MLP classifier.
RGAT [6]	Relational Graph Attention Network that leverages relation-aware attention to weight neighbor contributions across multiple edge types, with the learned node embeddings input to an MLP for downstream prediction.
GNN-FiLM [7]	Graph neural network with Feature-wise Linear Modulation layers that adaptively transform node features based on relation types, followed by an MLP module for prediction.
HAN [8]	Heterogeneous Graph Attention Network combining node-level attention and meta-path-level attention to capture semantic-aware node embeddings, which are then fed into an MLP for classification.
metapath2vec [9]	Meta-path-guided random walk model that learns node embeddings via skip-gram, with the resulting embeddings used as input to an MLP classifier.

Table S14. Input Features for Baseline Models

Model	Input feature		Brief description
HGT /<br>RGCN /<br>RGAT	9 node types:<br>psite<br>protein<br>pathway<br>molecular function<br>kinase<br>biological process<br>disease<br>gene<br>GO	10 edge types:<br>molecular function-psite<br>kinase-psite<br>biological process-psite<br>protein-psite<br>psite-psite<br>disease-disease<br>gene-disease<br>GO-disease<br>pathway-disease<br>gene-protein	A heterogeneous graph with nine node types and ten edge types is constructed following the same strategy as CDVGL-PDA. The graph is processed by HGT/RGCN/RGAT to learn informative node embeddings. The embeddings of the target psite and disease nodes are concatenated and fed into a multilayer perceptron for classification.
GNN-FiLM /<br>HAN /<br>metapath2vec	9 node types:<br>psite<br>protein<br>pathway<br>molecular function<br>kinase<br>biological process<br>disease<br>gene<br>GO	9 meta-paths:<br>psite-molecular function-psite<br>psite-biological process-psite<br>psite-protein-psite<br>psite-kinase-psite<br>psite-psite<br>disease-disease<br>disease-gene-disease<br>disease-pathway-disease<br>disease-GO-disease	A heterogeneous graph consisting of nine node types and nine predefined meta-paths is constructed, with node features extracted in the same strategy as CDVGL-PDA. The graph is processed by GNN-FiLM/HAN/metapath2vec to learn informative node embeddings. The embeddings of the target psite and disease nodes are concatenated and fed into a multilayer perceptron for classification.
MSF-CNN	5 networks:<br>psite sequence similarity<br>psite Gaussian interaction profile kernel similarity<br>disease semantic similarity<br>disease symptom similarity<br>disease Gaussian interaction profile kernel similarity		Random Walk with Restart is applied to the two psite similarity networks and the three disease similarity networks to learn psite and disease embeddings, respectively. The embeddings of the target psite and disease are concatenated and fed into a convolutional neural network for classification.
TabPFN /<br>AutoGluon	psite + disease		The input includes preprocessed psite and disease features, constructed following the same strategy as CDVGL-PDA. The features of the target psite and disease nodes are concatenated and fed into these models for classification.

Table S15. Performance and computational cost comparison of graph-based models on the psite-level split benchmark.

Model	AUC	AUPR	Params (M)	Train Time / Epoch (s)	Inference Time (ms / batch)	Peak GPU Memory (MB)
metapath2vec	0.7996	0.8235	0.03	0.02	10.48	21.36
HGT	0.8445	0.8713	27.14	0.36	96.12	777.92
RGCN	0.8579	0.8653	6.05	0.10	42.30	280.42
RGAT	0.8597	0.8817	6.35	0.25	80.17	285.99
HAN	0.8622	0.8814	127.71	0.41	146.11	3135.44
GNN-FiLM	0.8624	0.8849	9.28	0.17	64.56	381.43
CDVGL-PDA	0.8949	0.9163	34.06	0.46	166.78	1971.30

Table S16. Major innovations of CDVGL-PDA.

Rank	Major Innovation	Description
1	Heterogeneous graph construction	Construct a heterogeneous graph with 9 node types and 10 edge types to provide a biological context for learning representations of psites and diseases.
2	Multi-modal node feature initialization	Apply tailored encoding strategies for different nodes, including sequence-based encoding for psites and proteins, text-based encoding for diseases, and graph-based encoding for other nodes.
3	Dual heterogeneous graph encoders	Integrate RGAT and HGT to learn complementary structural and semantic representations from heterogeneous biological networks.
4	Contrastive learning with attention-based fusion	Align multi-view representations via contrastive learning and integrate them using attention mechanisms to obtain robust unified embeddings.

Table S17. Sensitivity analysis of similarity thresholds in the psite sequence similarity network and disease semantic similarity network on the psite-level split benchmark.

Psite similarity threshold	Disease similarity threshold	AUC	AUPR
0.5	0.2	0.8949	0.9163
0.4	0.2	0.8901	0.9124
0.6	0.2	0.8913	0.9147
0.5	0.1	0.8872	0.9106
0.5	0.3	0.8920	0.9153

Table S18. Performance comparison of different initial feature dimensions on the psite-level split benchmark.

Initial Feature Dimension	AUC	AUPR
64	0.8857	0.9120
128	0.8949	0.9163
256	0.8711	0.8973

Table S19. Performance comparison of different sequence lengths on the psite-level split benchmark.

Metric	71	51	33	15	21
AUC	0.8949	0.8928	0.8866	0.8908	0.8916
AUPR	0.9163	0.9189	0.9131	0.9154	0.9198

Table S20. Hyperparameters settings for CDVGL-PDA

**Hyperparameters**	**Search space**	**Best parameter**
Learning rate	[0.01, 0.001]	0.001
Layers of RGAT	[1, 2, 3]	2
Layers of HGT	[1, 2, 3]	2
Layers of GRU	[1, 2]	1
Temperature parameter	[0.4, 0.5, 0.6]	0.5
Dimensions of hidden layers of network	[256, 512, 1024]	512
Dropout rate	[0.1, 0.2, 0.3, 0.4]	0.2
Number of Attention heads in RGAT	[4, 8]	8
Number of Attention heads in HGT	[4, 8]	8
Weight decay	[0.001, 0.0001, 0.00001]	0.0001
Psite similarity threshold	[0.4, 0.5, 0.6]	0.5
Disease similarity threshold	[0.1, 0.2, 0.3]	0.2
λ_1 (psite contrastive loss)	[1, 5, 10, 15]	1
λ_2 (disease contrastive loss)	[1, 5, 10, 15]	1
λ_3 (BCE prediction loss)	[1, 5, 10, 15]	10

Table S21. Impact of loss weights (λ_1, λ_2, λ_3) on model performance.

λ_1(psite contrastive loss)	λ_2(disease contrastive loss)	λ_3(BCE prediction loss)	F1	AUC	AUPR
1	1	10	**0.8119**	**0.8949**	**0.9163**
5	5	10	0.8175	0.8807	0.9074
10	10	10	0.8157	0.8786	0.9064
15	15	10	0.8123	0.8785	0.9045
1	1	1	0.8156	0.8750	0.9032
1	1	5	0.8030	0.8860	0.9124
1	1	10	**0.8119**	**0.8949**	**0.9163**
1	1	15	0.8144	0.8909	0.9112
1	1	10	**0.8119**	**0.8949**	**0.9163**
1	5	10	0.8171	0.8916	0.9160
5	1	10	0.7924	0.8729	0.9013

Table S22. Model evaluation under protein-level splits with low sequence identity. Test sets were constructed from PDAs associated with approximately 10%, 20%, or 30% of proteins with the lowest maximum sequence identity.

proportion	Training pairs	Test pairs	AUC	AUPR
10%	7938	526	0.8947	0.8936
20%	7362	1102	0.8849	0.9018
30%	6866	1598	0.8906	0.9019

Table S23. Model evaluation under psite-level splits with low sequence identity. Test sets were constructed from PDAs associated with approximately 10%, 20%, or 30% of psites with the lowest maximum sequence identity.

proportion	Training pairs	Test pairs	AUC	AUPR
10%	7778	686	0.8949	0.9163
20%	7106	1358	0.9054	0.9201
30%	6434	2030	0.9029	0.9114

Table S24. Ablation study on phosphorylation site (psite) encoding strategies on the psite-level split benchmark.

Encoder	AUC	AUPR
BLOSUM62 + GRU	**0.8949**	**0.9163**
BLOSUM62	0.8880	0.9117
ESM2 + GRU	0.8848	0.9083
ESM2	0.8777	0.9018

Table S25. Data sources of node and edge types in the heterogeneous graph.

Category	Type	Data source
Node	psite	PSP[10]
Node	kinase	PSP
Node	protein	UniProt[11]
Node	disease	CTD[10]
Node	gene	CTD
Node	pathway	CTD
Node	molecular function	PSP
Node	biological process	PSP
Node	GO	CTD
Edge	molecular function–psite	PSP
Edge	biological process–psite	PSP
Edge	kinase–psite	PSP
Edge	protein–psite	PSP
Edge	gene–disease	CTD
Edge	GO–disease	CTD
Edge	pathway–disease	CTD
Edge	gene–protein	UniProt
Edge	psite–psite	Sequence similarity[12]
Edge	disease–disease	MeSHDAG-based semantic similarity[13]

Supplementary Texts:

Text S1. Hyperparameter optimization

To ensure optimal performance of CDVGL-PDA, we tuned key hyperparameters on the psite-level split benchmark, which poses a challenging evaluation scenario due to completely unseen psites in the test set and strict control of sequence identity. We performed a grid search over a predefined search space (Table S20) for parameters including learning rate (0.001), number of layers of RGAT (2), HGT (2), and GRU (1), hidden layer dimensions of network (512), number of attention heads in RGAT (8) and HGT (8), dropout rate (0.2), weight decay (0.0001), temperature parameter of contrastive learning (0.5), sequence similarity thresholds for psites (0.5) and diseases (0.2), and the loss weights λ_1 (1), λ_2 (1), and λ_3 (10).

Here, λ_1 and λ_2 correspond to the contrastive learning objectives for psite and disease nodes, respectively, while λ_3 controls the BCE loss for association prediction. As shown in Table S21, varying λ_1 and λ_2 from 1 to 15 leads to only modest changes in performance, indicating that CDVGL-PDA is relatively robust to the weighting of the contrastive objectives. However, excessively large contrastive loss weights slightly reduce AUC and AUPR, suggesting an overemphasis on representation alignment. For λ_3, performance improves as its value increases from 1 to 10, while a further increase to 15 results in a slight decline. This indicates that an appropriate balance between contrastive learning and association prediction is important for optimal performance. Overall, $\lambda_1 = 1$, $\lambda_2 = 1$, and $\lambda_3 = 10$ achieve the best performance (AUC = 0.8949, AUPR = 0.9163) and were therefore used in all subsequent experiments.

Text S2. Prediction performance stratified by node degree

To assess whether the proposed model is biased toward highly connected nodes, we evaluated prediction performance stratified by node degree. For each test association pair, we defined its total degree as the sum of the in-degrees of the corresponding psite and disease nodes, and stratified the test pairs into three groups based on the distribution of total degrees. The Low, Medium, and High groups correspond to the lowest 25%, middle 50%, and highest 25% of pairs, respectively. As shown in Table S6, model performance exhibits different degree-dependent patterns under the three evaluation settings, reflecting their distinct levels of difficulty.

Under the protein-level split, where sequence similarity between the proteins corresponding to test psites is strictly controlled, the AUC reaches 0.7515 for low-degree pairs, increases to 0.8896 for medium-degree pairs, and slightly decreases to 0.8533 for high-degree pairs. Notably, performance does not monotonically improve with node degree, indicating that the model does not simply favor highly connected nodes. Under the psite-level split, where test psites are entirely unseen during training and local sequence similarity is constrained, performance on low-degree pairs drops substantially (AUC = 0.6169), reflecting the increased difficulty of cold-start prediction under limited structural and relational information. However, performance improves markedly for medium- and high-degree pairs (AUC = 0.8909 and 0.9057, respectively), suggesting that sufficient neighborhood information can effectively compensate for the lack of sequence similarity. Under the pair-level split, where individual nodes may be observed during training but test pairs are constrained to be dissimilar from training pairs, the model maintains consistent performance across degree groups. The AUC is 0.7075 for low-degree pairs and increases to 0.8357 and 0.8285 for medium- and high-degree pairs, respectively. This demonstrates that the model retains discriminative ability even when direct pairwise similarity signals are minimized.

Overall, node degree influences prediction difficulty under different evaluation settings, while the model maintains reasonable performance across degree groups and is not solely dependent on highly connected nodes.

Text S3. Construction of psite Sequence Similarity and Disease Semantic Similarity Networks

For the psite sequence similarity network, we extracted a 71-residue sequence centered on each psite and computed pairwise sequence identity using the Needleman-Wunsch algorithm[12]. These identity values were directly used as similarity measures to construct the psite–psite similarity matrix, which serves as the adjacency matrix of the psite sequence similarity network.

For the disease semantic similarity network, the biological meaning of a disease is closely related to its associated disease terms, and semantic similarity between diseases can be calculated based on the similarity of their terms. We adopt Wang D's method[13] to compute disease semantic similarity. In MeSH database, a disease a can be represented as a directed acyclic graph $DAG_a = (T_a, E_a)$, where T_a denotes the set containing disease a and all its ancestor nodes, and E_a represents the edges describing the semantic relationships among the nodes in T_a. The semantic contribution of any disease $t_i \in T_a$ to disease a is defined as:

$$D_a(t_i) = \begin{cases} 1, & \text{if } t_i = a, \\ \max\limits_{t' \in children(t_i)} \left\{ \frac{1}{2} \cdot D_a(t') \right\}, & \text{if } t_i \neq a, \end{cases} \tag{S1}$$

where $children(t_i)$ denotes the set of child nodes of t_i, and $\frac{1}{2}$ is the semantic contribution weight of edge (t_i, t'). Then, the semantic similarity between diseases a and b is defined as:

$$Sim(a,b) = \frac{\sum_{t_i \in T_a \cap T_b} \left(D_a(t_i) + D_b(t_i)\right)}{DV(a) + DV(b)}. \tag{S2}$$

where $DV(a) = \sum_{t_i \in T_a} D_a(t_i)$ represents the total semantic value of a, and $DV(b) = \sum_{t_i \in T_b} D_b(t_i)$ represents the total semantic value of b. This formulation quantifies the semantic similarity of two diseases based on their hierarchical relationships in the DAG. Based on this method, we constructed a disease–disease semantic similarity matrix (referred to as MeSHDAG-based disease similarity matrix), a higher similarity value indicates that two diseases share more common ancestors and exhibit closer semantic meanings, suggesting potential overlaps in their pathological mechanisms or biological systems. This similarity matrix serves as the adjacency matrix of the disease semantic similarity network, representing the pairwise semantic similarities among all diseases.

Text S4. Uncovering disease hierarchical relationships after removing disease-disease edges

To verify whether the learned disease representations can reflect semantic associations among diseases, we retrained the model on a heterogeneous graph with all disease–disease edges removed, and subsequently extracted the embedding vectors of 293 disease nodes. Pairwise Euclidean distances were computed between these embeddings to construct a disease representation distance matrix, denoted as $\mathbf{D}^{\mathrm{Euc}}$, which quantifies the geometric dissimilarities among diseases.

To assess whether these geometric distances align with the semantic hierarchy of diseases, we further constructed a hierarchical distance matrix for these 293 diseases based on the MeSH database. Each disease is associated with one or more TreeNumbers, representing its position in the MeSH hierarchy (dot-delimited notation). For any two diseases d_i and d_j, we compared all possible pairs of their TreeNumbers and calculated the length of their longest common prefix (LCP), denoted as L_{ij}. Let l_i and l_j represent the total number of hierarchical levels of d_i and d_j, respectively. The MeSH-based hierarchical distance between them is then defined as:$D_{ij}^{\mathrm{MeSH}} = (l_i - L_{ij}) + (l_j - L_{ij})$.When multiple TreeNumbers exist for either disease, we selected the minimal D_{ij}^{MeSH} among all combinations as the final hierarchical distance, yielding a symmetric MeSH-based hierarchical distance matrix $\mathbf{D}^{\mathrm{MeSH}}$. We next counted the number of disease pairs at each MeSH hierarchical distance and plotted their distribution to ensure sufficient sample sizes across levels. Then, for each distance group, we calculated the mean and standard deviation of the Euclidean distances and visualized their trend with error bars.

The results (Fig.3b) show that the mean Euclidean distance between disease embeddings increases with MeSH hierarchical distance, while the standard deviation remains relatively stable. In addition, we computed the correlation between embedding-based cosine similarity and MeSHDAG-based semantic similarity after removing disease–disease edges. The results show a moderate positive correlation (Pearson = 0.290, Spearman = 0.501). This indicates that even without explicit disease–disease edges, the model is still able to capture hierarchical semantic relationships, such relationships can be inferred indirectly from the heterogeneous network.

Text S5. Uncovering psite sequence similarity relationships after removing psite–psite edges

To evaluate whether the learned representations of psites can reflect sequence similarity, we retrained the model on a heterogeneous graph with all psite–psite edges removed, and extracted the embedding vector of each psite. Pairwise Euclidean distances between psite embeddings were then computed to obtain the complete Euclidean distance matrix. The original psite similarity matrix was constructed from pairwise sequence identity calculated using the Needleman-Wunsch algorithm on 71-residue sequences centered on each psite. We divided all psite pairs into five intervals according to their original similarity scores, and for each interval, we calculated the number of pairs, as well as the mean and standard deviation of their Euclidean distances.

As shown in Fig.3c, the majority of psite pairs exhibit low similarity scores, corresponding to larger mean Euclidean distances. In contrast, pairs with high similarity scores are much fewer in number but display markedly smaller Euclidean distances, revealing a negative correlation between sequence similarity and embedding-space distance. These results indicate that the learned psite embeddings effectively discriminate between similar and dissimilar sequences in the latent space: similar sequences are positioned closer together, whereas dissimilar ones are farther apart. This embedding distribution suggests that the model successfully encodes the local contextual information of psites, integrating both structural signals from the heterogeneous graph and intrinsic sequence features. Even after the removal of direct psite–psite edges, the model can still infer latent relationships among psites through other relational and contextual pathways, demonstrating its capability for potential edge discovery and feature generalization.

Text S6. Correlation between number of shared psites and disease similarity

We enumerated all disease pairs and calculated the number of shared psites, resulting in 3,155 disease pairs that share at least one psite. The disease pairs were then grouped according to the number of shared psites. We evaluated disease similarity using both the embedding-based disease similarity and the MeSHDAG-based disease similarity.

As shown in Fig. S3a, the median and mean embedding-based similarities increase with the number of shared psites, indicating that diseases sharing more psites tend to exhibit higher embedding similarity. This suggests that diseases with greater overlap in psites may involve more similar molecular mechanisms. The scatter distribution shows that most disease pairs share only 1-5 psites, while pairs sharing more than 50 psites are rare, reflecting the sparsity of the PDA dataset. Similarly, Fig. S3b presents the MeSHDAG-based disease similarity results, where both the median and mean similarity values increase with the number of shared psites. The consistent upward trend across both measures indicates that diseases with greater psite overlap are not only closer in embedding space but also more similar in semantic space. Together, these results support the idea that psite overlap can serve as an informative indicator of disease relatedness and may reflect shared underlying molecular mechanisms.

Text S7. Predictive performance on Disease-Level benchmarks

To further evaluate the performance of our model on the PDA prediction task, we constructed two disease-level datasets for Alzheimer disease (AD) and colorectal neoplasms (CRC), respectively, for comparative analysis. Taking AD as an example, we first removed all known associations between AD and psites from the PDA dataset. The remaining PDA pairs, together with an equal number of randomly sampled negative samples, were used to construct the training set, which excluded any AD-related associations. For testing, the original AD-psite associations were retained as positive samples. Meanwhile, negative samples were generated by selecting unassociated pairs with the disease ID corresponding to AD from the negative pool, followed by random sampling to obtain an equal number of negative samples. This resulted in a balanced test set. The same procedure was applied to CRC. The constructed balanced test sets ensure a fair comparison among different methods.

As shown in Fig. S4, CDVGL-PDA demonstrates strong performance in predicting psite-disease associations. On the AD dataset, the model achieves an AUC of 0.7884 and an AUPR of 0.8282. On the CRC dataset, the AUC and AUPR reach 0.9617 and 0.9584, respectively, indicating competitive overall performance. These results suggest that CDVGL-PDA possesses robust predictive capability and generalization ability across different diseases.

Text S8. Network interpretability

To elucidate how the model extracts informative signals from a heterogeneous biological network, we perform interpretability analyses from two perspectives: a global semantics branch and a local attention branch. The former captures how heterogeneous semantic relations are weighted at a global scale, while the latter interprets attention assigned to neighboring nodes during aggregation. Reverse edges (e.g., protein–psite/psite–protein) are included during graph construction to enable bidirectional information flow.

For the global semantics branch, we analyze the message projection matrices of each meta-relation in the two HGT layers and quantify their strengths using Frobenius norms. Larger values indicate greater importance in information propagation. As shown in Fig.4a, several relations targeting psite nodes are strengthened in the second layer, such as kinase–psite (1.55 to 2.02) and process–psite (1.43 to 2.11), suggesting enhanced modeling of higher-order associations. In contrast, relations such as disease–pathway (0.41 to 0.01) are markedly attenuated, indicating reduced information flow toward pathway nodes and suppression of less informative signals. This pattern is consistent with the task objective, as the model prioritizes learning PDAs, thereby emphasizing information relevant to psite and disease nodes while naturally down-weighting propagation to other entities.

For the local attention branch, we examine attention weights from the first GATConv layer of RGAT, normalized within each relation type. As illustrated in Fig.4b, for psite P08581_Y1349, attention is distributed across neighbors under different relation types. Notably, among four associated kinases, Kinase Q04912 receives the highest weight (0.26), indicating a stronger contribution to representation learning. Overall, RGAT adaptively assigns relation-specific weights to neighbors, demonstrating its effectiveness in capturing local structural heterogeneity.

Text S9. Construction of MeSH-constrained Candidate Negative Sample Pool

To construct a biologically constrained candidate negative sample pool, we leveraged the hierarchical structure of the MeSH disease classification system at the second level. The procedure is described as follows.

1. **Complete disease classification set:** We first extracted the second-level MeSH Tree Numbers from all diseases in the known PDAs to construct the complete set of second-level disease labels L_{all}. For example, $L_{\text{all}} = \{\text{C04}, \text{C14}, \text{C20}\}$,

representing neoplasms (C04), cardiovascular diseases (C14), and immune system diseases (C20), among all second-level MeSH categories.

2. **Positive label set:** For each psite p, we collected its known associated disease set and aggregated the corresponding second-level MeSH labels to form the positive label set $L_{\text{pos}}(p)$. For example, if p is associated with diseases d_1 and d_2, and both have second-level labels in C04, then $L_{\text{pos}}(p) = \{\text{C04}\}$.

3. **Uncovered label set:** By removing $L_{\text{pos}}(p)$ from L_{all}, we obtained the set of second-level labels not covered by p, denoted as $L_{\text{neg}}(p)$. For example, if $L_{\text{all}} = \{\text{C04}, \text{C14}, \text{C20}\}$ and $L_{\text{pos}}(p) = \{\text{C04}\}$, then $L_{\text{neg}}(p) = \{\text{C14}, \text{C20}\}$.

4. **Candidate negative disease:** Diseases whose second-level labels belong to $L_{\text{neg}}(p)$ were selected to form the candidate negative disease set $\text{neg}(p)$. For example, diseases labeled as C14 or C20 (e.g., d_3, d_4) are considered semantically incompatible with p and form the candidate negative set, $\text{neg}(p) = \{d_3, d_4\}$.

5. **Final candidate negative pool:** Each psite p was paired with all diseases in $\text{neg}(p)$, and all such pairs were aggregated to form the final candidate negative sample pool.

Using this MeSH-constrained strategy, we constructed a biologically constrained candidate negative sample pool. This strategy provides a conservative approximation by reducing the inclusion of highly semantically similar positive-like pairs. Our analysis (SI Text S6) showed that diseases sharing more psites tend to exhibit higher disease similarity, supporting the use of MeSH disease relationships for negative sample construction. However, due to the intrinsic functional heterogeneity within MeSH categories, a psite may be associated with specific diseases within a category but not others, and conversely, may participate in multiple disease contexts across different branches. Therefore, the resulting negative pool cannot completely eliminate false negatives, but it reduces semantic similarity between positive and negative pairs and provides a biologically more plausible set of negative samples.

Text S10. Distribution of unique psites across the top 20 diseases

We analyzed the distribution of unique psites across the top 20 most frequent diseases. As shown in Table S9, neoplasm-related diseases account for a substantial fraction of the dataset, reflecting the intensive experimental study of phosphorylation in cancer. At the same time, the dataset is not exclusively driven by a single disease. Several non-neoplastic diseases are also well represented, such as Alzheimer disease (231 psites), Parkinson disease (95 psites), diabetes mellitus (90 psites) and cardiomyopathies (58 psites), indicating a relatively diverse disease coverage.

Text S11. Distribution of unique psites across the top 20 proteins

We analyzed the distribution of unique psites across proteins using all collected positive samples. Table S10 summarizes the top 20 proteins ranked by the number of associated unique psites. The protein with the highest coverage contains 42 psites, whereas most proteins are associated with substantially fewer psites. Although a small number of proteins exhibit relatively higher phosphorylation coverage, reflecting their extensive experimental characterization, the dataset is not excessively dominated by hub proteins. Notably, the top 10 proteins together account for only 7.62% of all psites, indicating that many psites are distributed across proteins with relatively low psite coverage.

Text S12. Exploring psite distribution across disease hierarchy

We mapped diseases to the hierarchical structure defined by MeSH to determine their positions within the disease semantic system. We then linked curated psites to their associated diseases to analyze their distribution across the disease hierarchy. Based on this, we selected four representative cases for visualization and analysis.

- Psites P27361_Y204 and P28482_Y187 are associated with Diabetes Mellitus, Type 2, and are also observed in association with its parent disease, Diabetes Mellitus. According to the disease hierarchy (Fig. S6a), Diabetes Mellitus, Type 2 is a child of Diabetes Mellitus. This case illustrates that psites associated with a specific disease subtype can also be observed at higher levels of the disease hierarchy, reflecting a parent–child distribution pattern.

- Psites P31947_S74, P04637_S20, and P12931_Y419 are associated with Breast Neoplasms and are also linked to the higher-level disease category Neoplasms. According to the disease hierarchy (Fig. S6b), Breast Neoplasms is a second-level child of Neoplasms. This observation shows that psites may span multiple levels within a disease category, indicating a hierarchical spread from specific subtypes to broader disease classes.

- Psite P10636-8_T217 has been reported to be associated with both Myocardial Infarction and Hypertension. In addition, P10636-8_T181 is also linked to these two diseases. According to the disease hierarchy (Fig. S6c), both diseases belong to the Vascular Diseases category but reside on different sub-branches. This case highlights that psites can be shared across diseases within the same higher-level category, even when they are located in different branches of the hierarchy.
- Both Parkinson Disease and Diabetes Mellitus have been reported to be associated with distinct psites, while also sharing common psites such as P10636_S396. In addition, P10637_S554 has been reported to be associated with both diseases. As shown in Fig. S6d, these two diseases belong to different MeSH categories (Nervous System Diseases and Endocrine System Diseases) but are linked through shared psites. This observation illustrates that psites may be distributed across different disease systems, forming cross-category connections.

These representative cases illustrate several common patterns of PDAs across the MeSH hierarchy, including parent–child relationships, intra-category sharing, and cross-category connections. Together, these observations provide a descriptive view of the distribution of psites across disease hierarchies and highlight the complexity of their associations, underscoring the importance of systematically modeling these relationships.

Text S13. Construction of Low-Similarity Benchmarks

To assess model generalizability, we constructed three low-similarity benchmarks with more stringent constraints (overview in SI Table S11):

- **Protein-Level Split.** All 4,232 positive pairs were divided into training and test sets by assigning psites according to their source proteins. Pairwise sequence identity between proteins was computed using the Needleman-Wunsch algorithm[12]. Proteins were ranked according to their maximum sequence identity to all other proteins, and the 10% with the lowest identity were selected as the test set (see SI Text S14 for sensitivity analyses of different threshold settings). The psites associated with these proteins, together with their corresponding diseases, were used to form the positive test pairs, while the remaining proteins and their associated psites constituted the training set. Negative pairs were sampled from the MeSH-constrained pool to match the number of positive pairs. Training and test negative samples were restricted to proteins present in the corresponding positive sets to avoid overlap of proteins and psites.
- **Psite-Level Split.** Using the same 4,232 positive pairs, samples were grouped according to psites represented by 71-residue sequences centered on modification sites. Pairwise sequence identity between psites was computed using the Needleman-Wunsch algorithm[12]. Psites were ranked based on their maximum sequence identity to all other psites, and the 10% with the lowest identity were selected as the test set. The psites and their associated diseases were used to construct the positive test pairs, while the remaining psites and their corresponding diseases formed the training set. Negative pairs were sampled from the MeSH-constrained pool to match the size of the positive set. Training and test negatives were restricted to the corresponding psite groups to prevent overlap.
- **Pair-Level Split.** We define the similarity between two association pairs as the average of the corresponding psite sequence similarity and disease semantic similarity scores (similarity calculations are described in SI Text S3). For each positive pair, we compute its maximum similarity to all other positive pairs. Positive pairs are then ranked according to these maximum similarity scores, and approximately 10% of the pairs with the lowest similarity are selected to construct the test set, while the remaining pairs are used as training positives. Negative samples are drawn from the MeSH-constrained pool of unknown PDA pairs. Negative pairs in the training set are randomly sampled, whereas those in the test set are further filtered to ensure that their maximum similarity to any training positive pair is below the same threshold.

Text S14. Sensitivity analysis of low-similarity benchmark construction

To evaluate whether the choice of threshold affects the construction of the low-similarity benchmark datasets, we performed a sensitivity analysis using different test-set proportions. For the protein-level benchmark, proteins were ranked according to their maximum sequence identity to all other proteins, and approximately 10%, 20%, or 30% of proteins with the lowest maximum sequence identity were assigned to the test set. Similarly, for the psite-level benchmark, psites were ranked by their maximum sequence identity to all other psites, and the lowest-similarity 10%, 20%, or 30% were used to construct the test set. The remaining samples were used for training in each case.

The evaluation results are summarized in Tables S22 and S23. Under protein-level splits, CDVGL-PDA achieved AUC values ranging from 0.8849 to 0.8947 and AUPR values ranging from 0.8936 to 0.9019. Under psite-level splits, the model achieved AUC values ranging from 0.8949 to 0.9054 and AUPR values ranging from 0.9114 to 0.9201. Despite differences

in the sizes of the training and test sets, model performance remained consistently stable across all threshold settings, with no systematic decline as the proportion of low-similarity proteins or psites increased.

These results indicate that the proposed model is robust to different threshold choices and that the evaluation outcomes are not sensitive to the specific 10% threshold adopted in the main study. Therefore, the low-similarity benchmark provides a reliable assessment of model generalization to previously unseen proteins and psites with low sequence similarity.

Text S15. Description of all baseline methods

All baseline methods were trained and evaluated on the same dataset splits to ensure a fair comparison. For AutoML methods, we utilized standard default settings and built-in optimization pipelines. For heterogeneous graph representation learning methods, we explored reasonable hyperparameter values to select configurations that achieved good performance, with the specific settings listed in Table S12. For MSF-CNN, we directly adopted the parameter settings reported in the original study. Model description, and input features are now documented in Table S13 and Table S14.

We also compared computational costs. Since the baseline methods span fundamentally different learning paradigms, a unified comparison across all models is challenging. Therefore, we focused on comparing CDVGL-PDA with other graph-based methods, as CDVGL-PDA also relies on graph structures and leverages similar topological information. We evaluated four metrics: the number of trainable parameters, average training time per epoch, inference time per batch, and peak GPU memory usage. As shown in Table S15, CDVGL-PDA achieves the best predictive performance among all graph-based models, with the highest AUC (0.8949) and AUPR (0.9163), indicating its superior capability in capturing complex associations between psites and diseases. In terms of computational cost, CDVGL-PDA requires a moderate number of parameters (34.06M), which is significantly lower than HAN (127.71M) but higher than lightweight models such as RGCN and RGAT. The training time per epoch (0.46 s) is comparable to other attention-based models, while the inference time (166.78 ms per batch) and GPU memory usage (1971.30 MB) are higher than most baselines. This increased computational cost is expected, as CDVGL-PDA integrates more expressive modeling components to capture both structural and semantic relationships in heterogeneous graphs. Despite this, the model demonstrates a favorable trade-off between performance and efficiency, achieving substantial gains in prediction accuracy without incurring excessive parameter overhead compared to the most resource-intensive baseline (HAN).

Text S16. Details of heterogeneous graph construction

We constructed a heterogeneous graph consisting of nine node types and ten edge types to systematically integrate diverse biological information related to psites and diseases. The source database or construction method for each node and edge type is summarized in Table S25. Detailed descriptions of the edge types, data sources, and feature construction procedures are provided below.

1. Edge types and data sources

To systematically integrate biological knowledge of psites and diseases, we collected eight types of association networks from multiple authoritative databases. From PSP, we obtained four association networks related to psites to describe their biological characteristics from multiple perspectives:

- Psite-Molecular Function: links psites to the molecular functions they regulate, capturing activity and potential regulatory roles.
- Psite-Biological Process: connects psites to biological processes at cellular, tissue, or organismal levels, revealing higher-level functional roles.
- Psite-Kinase: indicates which kinases catalyze phosphorylation, providing causal information for upstream regulatory inference.
- Psite-Protein: records the proteins containing the psites, enabling indirect connections among sites on the same protein.

From the Comparative Toxicogenomics Database[10], we obtained three disease-related networks capturing disease characteristics from multiple aspects:

- Disease-Gene: links diseases to pathogenic or susceptibility genes, reflecting their genetic basis.
- Disease-GO: connects diseases to GO molecular function terms, providing functional annotations for disease nodes.
- Disease-Pathway: associates diseases with signaling and metabolic pathways, revealing potential mechanisms at the systems level.

From UniProt[11], we obtained a protein–gene association network recording the relationships between proteins and genes, such as encoding or regulatory relationships. This network provides a bridge in the heterogeneous graph, enabling multi-hop modeling of psite–protein–gene–disease relationships.

To enhance graph connectivity and capture latent functional and semantic relationships, we constructed psite sequence and disease semantic similarity networks using the Needleman-Wunsch algorithm and a MeSHDAG-based semantic similarity method, respectively (details in SI Text S3).

Edges were generated as follows. For the eight association networks, edges connect all database-recorded pairs. In the psite sequence similarity network, edges connect psites with similarity greater than 50%, while in the disease semantic similarity network, edges connect diseases with similarity greater than 20%. These thresholds were treated as hyperparameters and chosen to balance graph sparsity and density (SI Table S17). The resulting heterogeneous graph contains 207,064 edges across ten edge types (Table 1) and provides a balanced topology for stable downstream modeling.

2. Node types and feature construction

The heterogeneous graph contains 15,937 nodes across nine types, each representing distinct biological semantics and data modalities. We constructed node features tailored to each type, with an initial feature dimension of 128, chosen to balance representational capacity and computational efficiency (SI Table S18). The graph encoders integrate these features into a unified manifold, enabling reasoning across diverse biological modalities.

For psite nodes, we extracted a 71-residue sequence centered on each site, with 35 residues on each side and padding with X where necessary. This length was selected based on comparisons with commonly used window sizes (15, 21, 33, 51, and 71[14–16]), which showed comparable performance, and 71 residues was adopted in this study (see SI Table S19). Sequences were encoded using the BLOSUM62 substitution matrix and fed into the Phosphorylation Site Encoder (Fig. 1c) to generate initial features. Further ablation studies and comparisons with alternative encoding strategies, including pretrained protein language models (ESM2), are provided in SI Text S17.

Disease nodes were encoded using BioBERT[17], a language model pre-trained on biomedical text to capture contextual semantics of disease names. The [CLS] hidden state was extracted and reduced to 128 dimensions via PCA. Protein and kinase nodes were represented using ESM2[18], a protein language model that captures latent structural and functional information from amino acid sequences. The 1280-dimensional embeddings were reduced to 128 dimensions using PCA. For pathway, molecular function, biological process, gene, and GO nodes, Node2vec[19] was applied to their respective networks. By performing biased random walks and learning embeddings with the Skip-gram model, Node2vec captures graph structural and functional relationships. Resulting embeddings were set to 128 dimensions as initial node features.

Text S17. Ablation study on psite encoding strategies

To evaluate the effectiveness of different psite encoding strategies, we conducted an ablation study on the psite-level split dataset. We compared four variants: BLOSUM62+GRU, BLOSUM62 alone, ESM2+GRU, and ESM2 alone. Specifically, for the ESM2-based variants, residue-level embeddings were first extracted from full-length protein sequences to preserve global context, and the feature vectors corresponding to the central psites were subsequently retrieved. As shown in Table S24, the BLOSUM62-based representations consistently outperformed their ESM2-based counterparts in both AUC and AUPR. Incorporating a GRU layer further boosted performance for both encoding schemes, confirming that explicitly modeling local sequential dependencies remains highly beneficial. The optimal performance was achieved by the BLOSUM62+GRU architecture.

These results suggest that while ESM2 provides rich representations, they do not necessarily outperform classical evolutionary features for highly localized prediction tasks. This likely stems from the nature of psite recognition, which is heavily governed by sharp, local sequence motifs. The explicit substitution scores from BLOSUM62, paired with a sequence-gating GRU, offer a more direct representation of these micro-environments. In contrast, the massive global context captured by ESM2 can sometimes dilute such highly localized catalytic signals. Consequently, classical substitution-based encoding combined with sequential modeling remains exceptionally competitive for psite-centric tasks.

References

[1] Qian Deng et al. Identifying protein phosphorylation site-disease associations based on multi-similarity fusion and negative sample selection by convolutional neural network. *Interdisciplinary Sciences: Computational Life Sciences*, 16(3):649–664, 2024.

[2] Noah Hollmann, Samuel Müller, et al. Accurate predictions on small data with a tabular foundation model. *Nature*, 637(8045):319–326, 2025.

[3] Nick Erickson, Jonas Mueller, et al. Autogluon-tabular: Robust and accurate automl for structured data. *arXiv preprint arXiv:2003.06505*, 2020.

[4] Ziniu Hu et al. Heterogeneous graph transformer. In *Proceedings of the web conference 2020*, pages 2704–2710, 2020.

[5] Michael Schlichtkrull et al. Modeling relational data with graph convolutional networks. In *European semantic web conference*, pages 593–607. Springer, 2018.

[6] Dan Busbridge et al. Relational graph attention networks. *arXiv preprint arXiv:1904.05811*, 2019.

[7] Marc Brockschmidt. Gnn-film: Graph neural networks with feature-wise linear modulation. In *International conference on machine learning*, pages 1144–1152. PMLR, 2020.

[8] Xiao Wang et al. Heterogeneous graph attention network. In *The world wide web conference*, pages 2022–2032, 2019.

[9] Yuxiao Dong et al. metapath2vec: Scalable representation learning for heterogeneous networks. In *Proceedings of the 23rd ACM SIGKDD international conference on knowledge discovery and data mining*, pages 135–144, 2017.

[10] Allan Peter Davis et al. Comparative toxicogenomics database's 20th anniversary: update 2025. *Nucleic acids research*, 53(D1):D1328–D1334, 2025.

[11] UniProt. Uniprot: the universal protein knowledgebase in 2025. *Nucleic acids research*, 53(D1):D609–D617, 2025.

[12] Saul B Needleman and Christian D Wunsch. A general method applicable to the search for similarities in the amino acid sequence of two proteins. *Journal of molecular biology*, 48(3):443–453, 1970.

[13] Dong Wang et al. Inferring the human microrna functional similarity and functional network based on microrna-associated diseases. *Bioinformatics*, 26(13):1644–1650, 2010.

[14] Renfei Ma et al. Kinasephos 3.0: redesign and expansion of the prediction on kinase-specific phosphorylation sites. *Genomics, proteomics & bioinformatics*, 21(1):228–241, 2023.

[15] Palistha Shrestha et al. Dl-sphos: Prediction of serine phosphorylation sites using transformer language model. *Computers in Biology and Medicine*, 169:107925, 2024.

[16] Jingxin Xie et al. Deepmpsf: A deep learning network for predicting general protein phosphorylation sites based on multiple protein sequence features. *Journal of Chemical Information and Modeling*, 63(22):7258–7271, 2023.

[17] Jinhyuk Lee et al. Biobert: a pre-trained biomedical language representation model for biomedical text mining. *Bioinformatics*, 36(4):1234–1240, 2020.

[18] Zeming Lin et al. Evolutionary-scale prediction of atomic-level protein structure with a language model. *Science*, 379(6637):1123–1130, 2023.

[19] Aditya Grover and Jure Leskovec. node2vec: Scalable feature learning for networks. In *Proceedings of the 22nd ACM SIGKDD international conference on Knowledge discovery and data mining*, pages 855–864, 2016.
